# Increased serum extracellular vesicle miR-144-3p and miR-486a-3p in a mouse model of adipose tissue regeneration promote hepatocyte proliferation by targeting *Txnip*

**DOI:** 10.1371/journal.pone.0284989

**Published:** 2023-05-04

**Authors:** Yoshihiro Niitsu, Chikara Komiya, Akira Takeuchi, Kazunari Hara, Masato Horino, Jun Aoki, Rei Okazaki, Masanori Murakami, Kazutaka Tsujimoto, Kenji Ikeda, Tetsuya Yamada

**Affiliations:** Department of Molecular Endocrinology and Metabolism, Graduate School of Medical and Dental Sciences, Tokyo Medical and Dental University, Tokyo, Japan; Lewis Katz School of Medicine, Temple University, UNITED STATES

## Abstract

Adipose-derived stem cells are expected to be applied to regenerative medicine for various incurable diseases including liver cirrhosis. Although microRNAs contained in extracellular vesicles (EV-miRNAs) have been implicated in their regenerative effects, the precise mechanism has not been fully elucidated. Tamoxifen-inducible adipocyte-specific insulin receptor knockout (iFIRKO) mice are known to exhibit acute adipose tissue regeneration with increased numbers of adipose stem and progenitor cells (ASPCs). Because adipose tissue is the major source of circulating EV-miRNAs, we investigated alterations in serum EV-miRNAs in iFIRKO mice. A comprehensive analysis using miRNA sequencing on serum EVs revealed that most EV-miRNAs were decreased due to the loss of mature adipocytes, but there were 19 EV-miRNAs that were increased in the serum of iFIRKO mice. Among them, miR-144-3p and miR-486a-3p were found to be increased in the liver as well as serum EVs. While the expression levels of pri-miR-144-3p and pri-miR-486a-3p were not increased in the liver, they were elevated in the adipose tissue, suggesting that these miRNAs may be delivered from ASPCs increased in the adipose tissue to the liver via EVs. Increased hepatocyte proliferation was observed in the liver of iFIRKO mice, and we found that both miR-144-3p and miR-486a-3p have a function to promote hepatocyte proliferation by suppressing *Txnip* expression as a target gene. miR-144-3p and miR-486a-3p can be candidate therapeutic tools for conditions requiring hepatocyte proliferation, such as liver cirrhosis, and our current study suggests that examining EV-miRNAs secreted *in vivo* may lead to the discovery of miRNAs involved in regenerative medicine that have not been identified by *in vitro* analysis.

## Introduction

Adipose tissue is now widely recognized as an endocrine organ as well as an energy storage organ, and many studies have reported that adipokines play pivotal roles in physiological and pathological inter-organ crosstalk [1, 2]. Excessive lipid accumulation in white adipose tissue (WAT) due to obesity, for example, causes altered adipokine secretion. In addition, hypersecretion of free fatty acids, leptin, and inflammatory cytokines from the adipose tissue is strongly involved in the development of non-alcoholic steatohepatitis (NASH) [3]. Brown adipose tissue (BAT) also secretes adipokines called batokines, such as fibroblast growth factor 21 (FGF21), which is secreted under cold condition and enhances systemic energy expenditure through thermogenesis by WAT beiging [4].

Recent studies have demonstrated that extracellular vesicles (EVs) are emerging mediators in intercellular and inter-organ communication [5, 6]. EVs are 30−1000 nm in size and are composed of lipid bilayers; they are released from almost all types of cells, containing a variety of proteins, lipids, and nucleic acids as cargos [5, 7]. Secreted EVs are taken up by other cells, and cargo molecules have a wide range of functions in the recipient cells [8]. Among the cargos of EVs, microRNAs (miRNAs) have attracted attention as they are currently known to be involved in the development and progression of various diseases, such as cancer, cardiovascular disease, and metabolic disorders [9,10,11]. miRNAs are single-stranded non-coding RNAs consisting of around 22 bases; they regulate gene expression by inhibiting the translation or degradation of mRNAs that have sequences complementary to miRNAs in the 3’-untranslated region (3’-UTR) [12, 13]. At present, many investigators have a strong interest in miRNAs contained in EVs (EV-miRNAs) secreted from adipose tissue. This is because recent research using adipose tissue-specific knockout mice of Dicer, an enzyme essential for miRNA processing, has demonstrated that adipose tissue is the major source of circulating EV-miRNAs in the blood [14]. This previous work also showed that some of the EV-miRNAs secreted from the adipose tissue are delivered to the liver, including miR-99b, which regulates the hepatic expression of its target gene *Fgf21* [14].

Adipose tissue is composed of many cell types, including adipocytes, endothelial cells, and immune cells, and each cell releases characteristic EV-miRNAs under certain circumstances. For instance, adipose tissue macrophages secrete more miR-155-containing EVs in obese mice than in lean mice, and miR-155 incorporated into insulin target organs impairs insulin sensitivity by repressing its target gene *Pparg* [15]. Mature adipocytes are differentiated from adipose stem and progenitor cells (ASPCs), and the contents of EVs secreted from ASPCs exhibit profound changes during differentiation processes [16−18]. It has been reported that mesenchymal stem cells (MSCs), including adipose-derived stem cells exert a therapeutic effect on tissue injury. It has also been elucidated that EVs secreted from these cells are responsible for most of this effect [19]. For example, MSC-derived EVs containing miR-494 have been shown to accelerate skeletal muscle regeneration in a mouse model of cardiotoxin-induced muscle injury [20], and miR-125b transferred to the liver via MSC-derived EVs has been reported to ameliorate liver fibrosis in a rat model of carbon tetrachloride-induced liver injury [21].

Because ASPCs are relatively easy to isolate, they are expected to be applied to regenerative therapy for various diseases including liver cirrhosis. Many studies have investigated EV-miRNAs secreted from ASPCs *in vitro* [22]; however, little is known about EV-miRNAs secreted *in vivo*. This is because ASPCs are minor populations in adipose tissue, making it difficult to analyze EV-miRNAs secreted from ASPCs separately from other cells. Thus, the molecular mechanism of the regenerative action caused by EVs has not been fully elucidated in most target diseases. To overcome this challenge, we focused on tamoxifen-inducible adipocyte-specific insulin receptor knockout (iFIRKO) mice and investigated the function of serum EV-miRNAs. iFIRKO mice exhibit dynamic adipose tissue regeneration accompanied by loss of pre-existing adipocytes due to abrupt lack in insulin signaling and emergence of newly differentiated adipocytes with increased numbers of ASPCs [23].

Here, we demonstrate that the profile of serum EV-miRNAs is dramatically altered in iFIRKO mice and that serum EVs isolated from these mice exert proliferative effects on hepatocytes. In addition, we demonstrate that miR-144-3p and miR-486a-3p would be delivered to the liver via serum EVs in iFIRKO mice and that both miRNAs have a function to promote hepatocyte proliferation by suppressing *Txnip* gene expression. Our data suggest that miRNAs with regenerative effects may be packaged in EVs and released into the bloodstream during acute adipose tissue regeneration with increased ASPCs and that miR-144-3p and miR-486a-3p can be candidate therapeutic tools for conditions requiring hepatocyte proliferation, such as acute liver failure and liver cirrhosis.

## Materials and methods

### Animals

All animal experiments were approved by Tokyo Medical and Dental University Committee on Animal Research (A2021-123C3, G2018-055C3). This study was conducted in accordance with the Fundamental Guidelines for Proper Conduct of Animal Experiment and Related Activities in Academic Research Institutions under the jurisdiction of the Ministry of Education, Culture, Sports, Science and Technology of Japan. C57BL/6J wild-type mice were purchased from CLEA Japan, Inc., and Adipoq-CreERT2 (stock number: 025124) and Insr (IR) flox/flox (stock number: 006955) mice from The Jackson Laboratory. We created tamoxifen-inducible adipocyte-specific insulin receptor knockout (iFIRKO) mice by crossing Adipoq-CreERT2 mice with IR flox/flox mice. The animals were housed at 25°C with a 12-h light/dark cycle and allowed free access to water and a standard diet (CE-2; 343 kcal/100 g, CLEA Japan, Inc.). Seven-week-old male iFIRKO or IR flox/flox (control) mice were intraperitoneally injected with 100 mg/kg tamoxifen (Sigma-Aldrich) for 5 consecutive days and were analyzed 3 days after the last administration of tamoxifen. Blood glucose was measured using StatStrip Xpress (Nova Biomedical). Serum insulin concentration was determined using an enzyme-linked immunosorbent assay kit (Morinaga Institute of Biological Science, Inc.).

### Histological analysis

The adipose tissue and the liver were fixed with 4% paraformaldehyde and embedded in paraffin. The sections were stained with hematoxylin and eosin (HE). The proliferative cells and the hepatocytes in the liver were detected immunohistochemically using Ki-67 (14-5698-82, Thermo Fisher Scientific Inc.) and HNF4α (3113, Cell Signaling Technology) antibodies, respectively. Ki-67 and HNF4α double-positive and HNF4α single-positive cells were counted in the whole area of the sections using ImageJ software.

### RNA isolation and quantitative RT-PCR

Total RNA was isolated using miRNeasy Mini Kit or QIAzol reagent (Qiagen). mRNA was reverse-transcribed with Random Primer (Thermo Fisher Scientific Inc.) and ReverTra Ace (Toyobo Co., Ltd.), and miRNA and primary miRNA (pri-miRNA) were reverse-transcribed using TaqMan MicroRNA Reverse Transcription Kit and High-Capacity cDNA Reverse Transcription Kit with RNase Inhibitor (Thermo Fisher Scientific Inc.), respectively. Quantitative PCR was performed using QuantStudio 6 Flex Real-Time PCR System with Fast SYBR Green Master Mix Reagent or TaqMan Fast Advanced Master Mix (Thermo Fisher Scientific Inc.). The primer sequences are presented in S1 Table. Data were normalized to the levels of *36b4* for mRNA, U6 snRNA for miRNA, or *Gapdh* for pri-miRNA, and were analyzed using the comparative CT method.

### mRNA sequencing analysis

cDNA libraries were generated from the total RNA of the liver using TruSeq stranded mRNA Library Prep Kit and were sequenced on the NovaSeq 6000 platform (Illumina) in a laboratory in DNA Chip Research, Inc. Differentially expressed genes were determined by fold change (>1.5), and gene ontology analysis was conducted using DAVID Bioinformatics Resources 6.8.

### miRNA sequencing analysis

The total RNA of EVs was isolated from the serum using miRCURY Exosome Serum/Plasma Kit and miRNeasy Micro Kit (Qiagen). cDNA libraries were prepared from pooled serum samples from seven mice using QIAseq miRNA Library Kit (Qiagen) and sequenced on the NextSeq500 platform (Illumina) in DNA Chip Research Inc. The sequencing data were analyzed on GeneGlobe Data Analysis Center (Qiagen). Differentially expressed genes were determined by fold change (>1.5), and validation studies were conducted by quantitative RT-PCR for each serum sample using TaqMan Advanced miRNA Assays and TaqMan Fast Advanced Master Mix (Thermo Fisher Scientific Inc.). Data were normalized to the levels of spike-in control cel-miR-39-3p or miR-148a-3p, a GeNorm algorithm-selected reference gene from the miRNA sequencing data.

### Western blotting

The liver and primary cultured hepatocytes were lysed in a lysis buffer (2% SDS, 4 M Urea, 1 mM EDTA, 150 mM NaCl, 50 mM Tris pH 8.0) supplemented with Halt Protease and Phosphatase Inhibitor Cocktail (Thermo Fisher Scientific Inc.). Immunoblotting was performed with CD9 (ab92726, Abcam Plc), TSG101 (ab125011, Abcam Plc), TXNIP (ab188865, Abcam Plc), and GAPDH (2118, Cell Signaling Technology) antibodies. Immunoblots were detected and analyzed using ECL Prime Western Blotting Detection Reagent and ImageQuant LAS 4000 mini (GE Healthcare).

### Cell culture and transfection

Hepa 1–6 cells were purchased from the American Type Culture Collection. Primary hepatocytes were isolated as previously described [24]. The cells were cultured in a humidified incubator at 37°C and 5% CO_2_ in Dulbecco’s Modified Eagle Medium (Nacalai Tesque Inc.) supplemented with 10% fetal bovine serum (Sigma-Aldrich) with or without 1−10 nM of human insulin (Eli Lilly and Company). miR-144-3p, miR-486a-3p, or Negative Control #1 mirVana mimics and Silencer Select siRNA targeting Txnip and Negative Control #1 were purchased from Thermo Fisher Scientific, Inc. Primary cultured hepatocytes were transfected with 10 nM of miRNA mimics or siRNA using Lipofectamine RNAiMAX Transfection Reagent (Thermo Fisher Scientific, Inc.) and were incubated for 36–48 h. For detection of cell proliferation, primary hepatocytes were immunocytochemically stained with Ki-67 antibody (14-5698-82, Thermo Fisher Scientific Inc.). For live cell counting, primary hepatocytes were quantified with Cell Counting Kit-8 (Dojindo).

### EV isolation

Serum samples were ultracentrifuged twice at 100,000 g at 4°C for 70 min, and the pellets were resuspended with phosphate-buffered saline (PBS). The EV size and concentration were analyzed using NanoSight LM10 (Malvern Panalytical Ltd.) in a laboratory in FUJIFILM Wako Pure Chemical Corporation.

### EV uptake assay

The isolated EVs were dyed using a PKH26 membrane labeling kit (Sigma-Aldrich) and were then washed four times on Amicon Ultra centrifugal filters (Merck Millipore) with PBS. Primary cultured hepatocytes were treated with 1 × 10^7^ particles/mL of PKH26-labeled EVs and incubated for 24 h. EVs incorporated into hepatocytes were detected using a confocal microscope (FLUOVIEW FV3000, Olympus Life Science) after washing with PBS and staining of nuclei with DAPI (Vector Laboratories).

### Dual-luciferase 3’-UTR reporter assay

The full-length *Txnip* 3’-UTR was amplified and cloned into the pmirGLO vector (Promega Corporation). The Hepa 1–6 cells were co-transfected with 0.2 μg of the vector and 100 nM of miR-144-3p, miR-486a-3p or Negative Control #1 mirVana mimics using Lipofectamine RNAiMAX Transfection Reagent and were then incubated for 48 h. The activity of Firefly luciferase normalized to that of Renilla luciferase was measured using Dual-Luciferase Reporter Assay System (Promega Corporation).

### Statistical analysis

Data were expressed as mean ± standard error of the mean and were compared using unpaired t-test for the comparison of two groups or analysis of variance with Tukey’s multiple comparisons test for the comparison of more than three groups. *p* < 0.05 was considered to be statistically significant. Statistical analysis was conducted using GraphPad Prism 9 (GraphPad Software, Inc.).

## Results

### Hepatocyte proliferation is induced in the liver of iFIRKO mice

**Fig 1:**
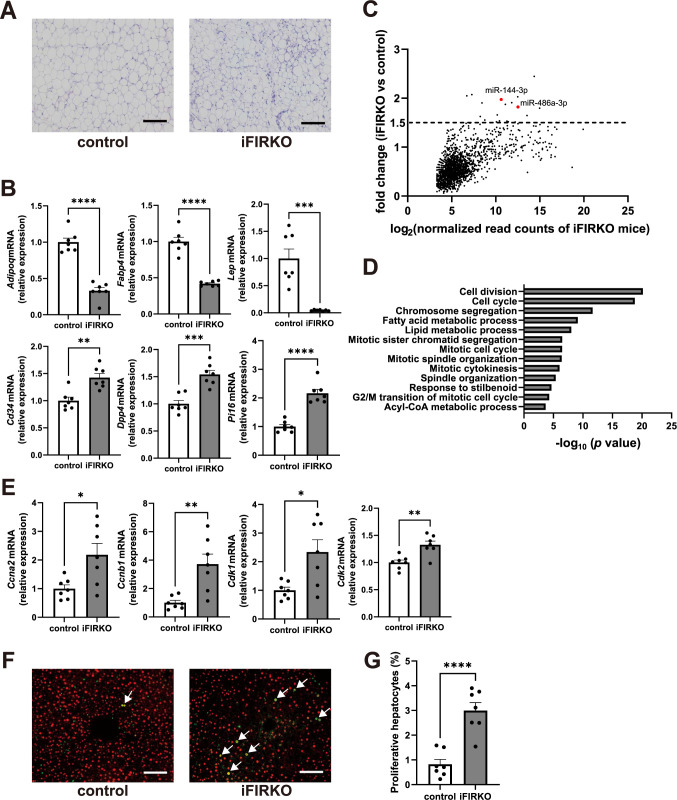
Alterations in serum EV-miRNAs and proliferation of hepatocytes are observed in iFIRKO mice. Seven-week-old male iFIRKO or IR flox/flox (control) mice were intraperitoneally injected with 100 mg/kg tamoxifen for 5 consecutive days and were analyzed 3 days after the last tamoxifen administration. (A) Representative images of HE staining of the inguinal WAT. (B) mRNA levels of adipocyte and ASPC marker genes in the inguinal WAT. (C) Plots displaying the fold change versus log_2_ normalized read counts of miRNA sequencing on serum EVs of iFIRKO mice compared with control mice. Red dots indicate miR-144-3p and miR-486a-3p. (D) Gene ontology enrichment analysis for the upregulated (>1.5-fold) genes in the liver of iFIRKO mice compared with the control mice. (E) Gene expression levels in the liver related to cell proliferation. (F) Representative images of Ki-67 (green) and HNF4α (red) immunostaining of the liver. Arrows indicate Ki-67 and HNF4α double-positive cells. (G) Quantification of proliferative hepatocytes in the liver. Original magnification, ×200. Scale bars, 100 μm. *p < 0.05, **p < 0.01, ***p < 0.001, ****p < 0.0001. *n* = 7.

To investigate the changes in EV-miRNAs during the rapid elimination of mature adipocytes, a major source of circulating EV-miRNAs [14], we used iFIRKO mice. As previously reported [23], 3 days after tamoxifen administration, the iFIRKO mice exhibited severe lipodystrophy with insulin resistance (S1A–S1E Fig), and the adipose tissue of iFIRKO mice was being regenerated with increased stromal cells (Fig 1A, S1F and S1G Fig). Gene expression analysis of the inguinal WAT in the iFIRKO mice revealed decreased levels of adipocyte marker genes, such as *Adipoq*, *Fabp4*, and *Lep*, and increased levels of stem and progenitor cell marker genes [17], such as *Cd34*, *Dpp4*, and *Pi16*, compared with the control mice (Fig 1B). We conducted miRNA sequencing analysis on serum EVs isolated from iFIRKO and control mice and found that most miRNAs were decreased in iFIRKO mice, reflecting the loss of mature adipocytes (Fig 1C). Of note, 19 EV-miRNAs were increased more than 1.5-fold in the serum of iFIRKO mice compared with the control mice, indicating that they may have been released into the bloodstream during the acute adipose tissue regeneration with increased ASPCs (Fig 1C).

We then examined the gene expression changes in the liver of iFIRKO mice, as adipose tissue-derived miRNAs have been reported to be delivered to liver via EVs and regulate gene expression [14]. RNA sequencing analysis on the liver revealed that 635 genes were upregulated by more than 1.5-fold in iFIRKO mice than in the control mice, and the gene ontology analysis of these genes was strongly associated with cell proliferation (Fig 1D). Quantitative RT-PCR assays demonstrated a significant increase in the expression of cell cycle-promoting genes, such as *Ccna2*, *Ccnb1*, *Cdk1*, and *Cdk2*, in the liver of iFIRKO mice compared with control mice (Fig 1E). Furthermore, liver immunohistochemistry revealed that the number of Ki-67-positive cells was increased in iFIRKO mice and that the majority of Ki-67-positive cells were merged with HNF4α, indicating that hepatocyte proliferation was enhanced in the liver of iFIRKO mice (Fig 1F and 1G).

### Serum EVs of iFIRKO mice promote cell proliferation in primary cultured hepatocytes

**Fig 2:**
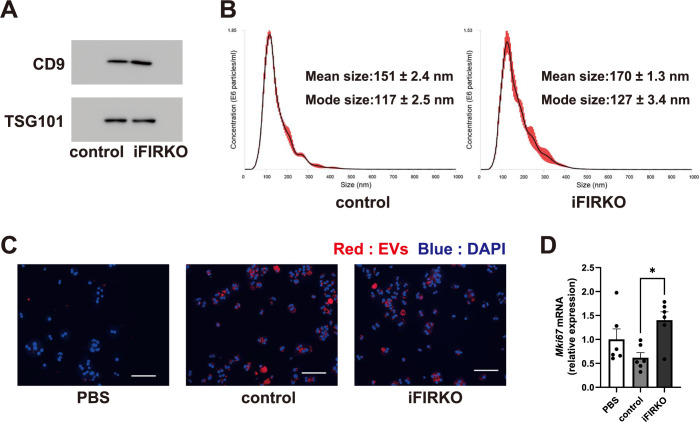
Serum EVs of iFIRKO mice promote hepatocyte proliferation *in vitro*. (A) Representative immunoblots of EV markers against EVs isolated from the serum of iFIRKO and control mice. (B) Size and concentration profiles of isolated EVs measured by nanoparticle tracking analysis. (C) Representative images and (D) *Mki67* mRNA levels of primary cultured hepatocytes treated with 1 × 10^7^ particles/mL of PKH26-labeled EVs (red) or PBS containing PKH26 dye. Blue: DAPI. Original magnification, ×200. Scale bars, 100 μm. *p < 0.05. *n* = 6.

Since hyperglycemia, hyperinsulinemia, or potential changes in other humoral factors may affect hepatocyte proliferation in iFIRKO mice, we next determined whether serum EVs of iFIRKO mice are actually involved in hepatocyte proliferation. We obtained EVs from the serum of iFIRKO and control mice and added them to primary cultured hepatocytes of wild-type mice. Western blotting confirmed that the ultracentrifugation-purified serum EVs were positive for the EV markers CD9 and TSG101 (Fig 2A), and nanoparticle tracking analysis revealed comparable EV concentrations and size distributions between the iFIRKO and control mice (Fig 2B). Fluorescence microscopy imaging showed that PKH26-labeled serum EVs isolated from iFIRKO and control mice appeared to be incorporated into perinuclear area of hepatocytes to the same extent (Fig 2C). Intriguingly, hepatocytes treated with serum EVs of iFIRKO mice exhibited significantly higher levels of *Mki67* expression than those treated with serum EVs of control mice (Fig 2D). These observations indicate that the differences in the serum EV cargos between iFIRKO and control mice affect hepatocyte proliferation.

### miR-144-3p and miR-486a-3p would be delivered to the liver via serum EVs in iFIRKO mice

**Fig 3:**
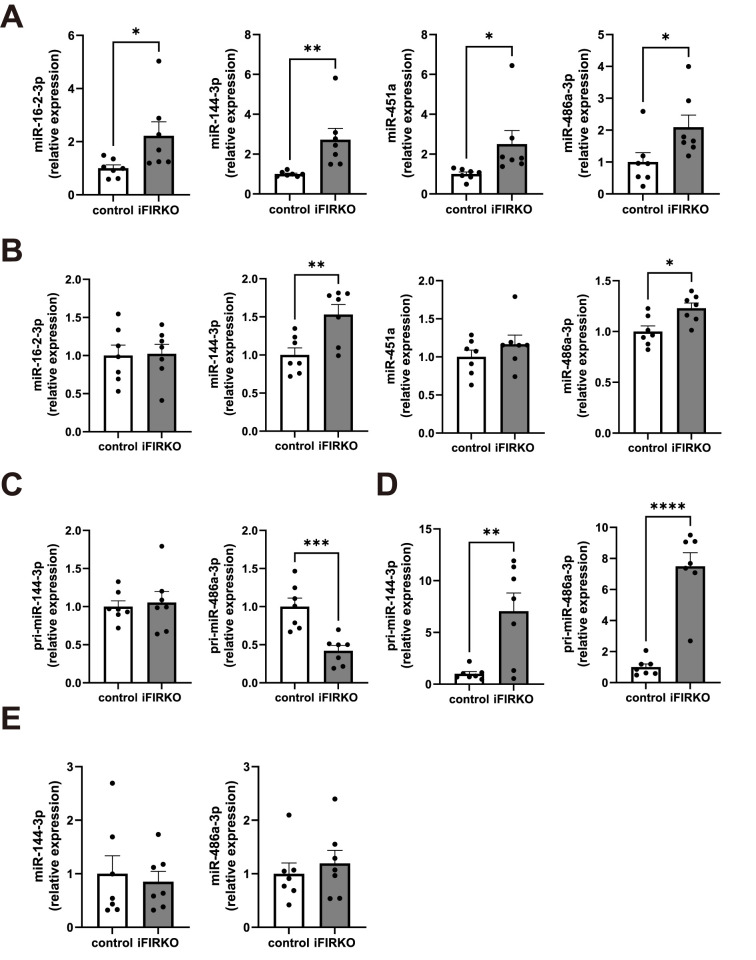
miR-144-3p and miR-486a-3p would be delivered to the liver via serum EVs in iFIRKO mice. (A) Increased serum EV-miRNAs validated by quantitative RT-PCR. Data were normalized to the levels of miR-148a-3p, which is a GeNorm algorithm-selected reference gene from the miRNA sequencing data. (B) Mature miRNA levels in the liver. Expression levels of pri-miR-144-3p and pri-miR-486a-3p in (C) the liver and (D) the inguinal WAT. (E) Levels of mature miR-144-3p and miR-486a-3p in the inguinal WAT. *p < 0.05, **p < 0.01, ***p < 0.001, ****p < 0.0001. *n* = 7.

We then explored miRNAs that were both increased in the serum EVs and liver of iFIRKO mice, as miRNAs delivered to the liver via circulating EVs may promote hepatocyte proliferation. Of the 19 EV-miRNAs found to be increased in the serum of iFIRKO mice by miRNA sequencing analysis, miR-16-2-3p, miR-144-3p, miR-451a, and miR-486a-3p were validated by quantitative RT-PCR assays for individual serum sample (Fig 3A, S2 Fig). Quantitative analysis of these miRNAs in the liver revealed that miR-144-3p and miR-486a-3p were significantly increased in iFIRKO mice compared with the control mice (Fig 3B). Importantly, the expression level of pri-miR-144-3p was not elevated and that of pri-miR-486a-3p was rather reduced in the liver of iFIRKO mice (Fig 3C). Mature miRNAs are generated from primary miRNAs through multiple steps of processing and can be released out of cells [25]. Because primary miRNAs are transcripts of miRNA genes and are localized in the nucleus of the original cell, the discrepancy in the amounts of mature miRNA and primary miRNA suggests that there is an influx or efflux of mature miRNAs in the cell [25]. The expression levels of pri-miR-144-3p and pri-miR-486a-3p were strongly elevated in the adipose tissue of iFIRKO mice, except for pri-miR-486a-3p in the epididymal WAT (Fig 3D, S3A Fig). Notably, mature forms of both miRNAs were not increased in the adipose tissue of iFIRKO mice (Fig 3E, S3B Fig). Taken together, these results suggest that miR-144-3p and miR-486a-3p would be delivered to the liver via serum EVs in iFIRKO mice, with the adipose tissue being a possible origin.

### miR-144-3p or miR-486a-3p mimic promotes cell proliferation in primary cultured hepatocytes

**Fig 4:**
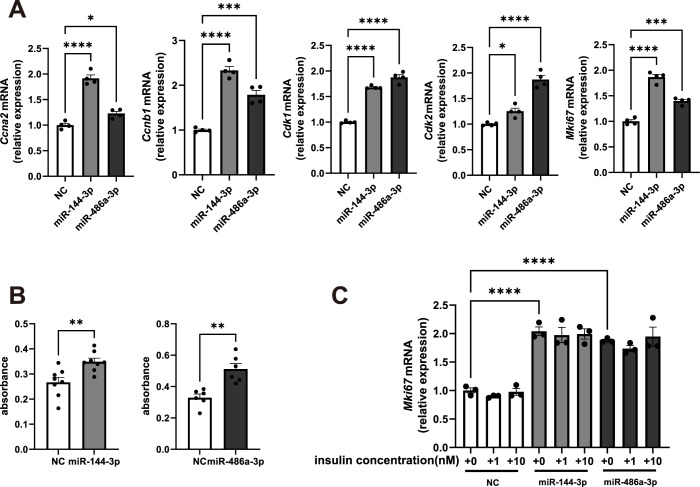
Transfection of miR-144-3p or miR-486a-3p mimics into hepatocytes results in cell proliferation *in vitro*. Primary cultured hepatocytes were transfected with 10 nM of negative control (NC), miR-144-3p, or miR-486a-3p mimics. (A) Gene expression levels related to cell proliferation. (B) Cell proliferation assessed using a live cell counting kit. (C) *Mki67* mRNA levels at increasing insulin concentrations in the medium. *p < 0.05, **p < 0.01, ***p < 0.001, ****p < 0.0001. *n* = 3−6.

To determine whether miR-144-3p and miR-486a-3p that were increased in the liver of iFIRKO mice contribute to hepatocyte proliferation, we conducted transfection experiments with primary cultured hepatocytes using miRNA mimics. Compared with the negative control miRNA mimics, the transfection of miR-144-3p or miR-486a-3p mimics into hepatocytes showed significant elevations in the expression of cell cycle-promoting genes, such as *Ccna2*, *Cdk1*, *Cdk2*, and *Mki67*, as observed in the liver of iFIRKO mice (Fig 4A). Cell proliferation was confirmed by Ki-67 immunostaining (S4 Fig) and a live cell counting assay (Fig 4B) 48 h after the transfection of miR-144-3p or miR-486a-3p mimics into the hepatocytes. Because the serum levels of insulin were increased in iFIRKO mice (S1D Fig), we changed the insulin concentration in the culture medium and conducted the same transfection study. However, the insulin concentration in the culture medium did not affect the hepatocyte proliferation caused by the transfection of miR-144-3p or miR-486a-3p mimics (Fig 4C).

### *Txnip* is a direct target of miR-144-3p and miR-486a-3p, and hepatocyte proliferation in the liver of iFIRKO mice would be induced by suppressing *Txnip* expression

**Fig 5:**
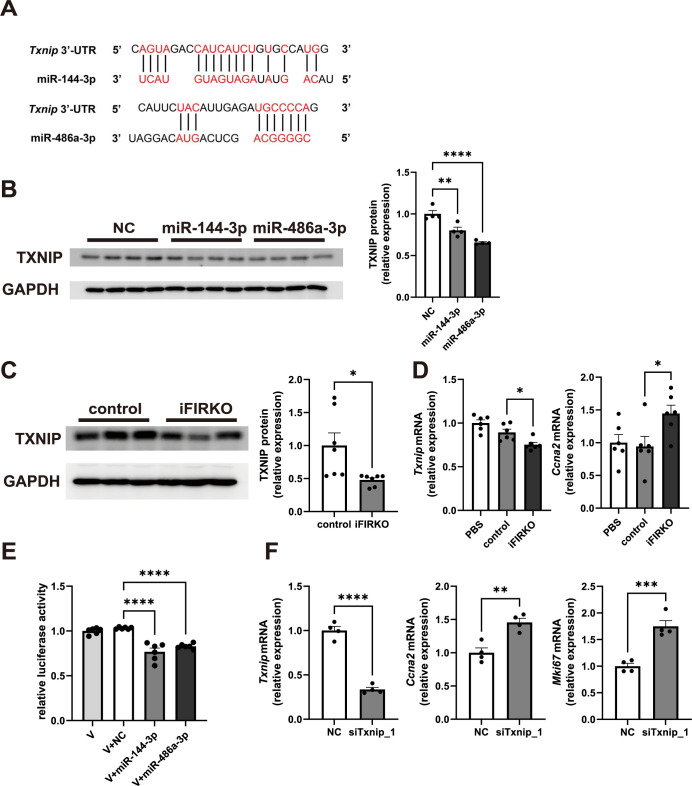
miR-144-3p and miR-486a-3p promote hepatocyte proliferation by directly targeting *Txnip*. (A) *Txnip* 3’-UTR target sequences of miR-144-3p and miR-486a-3p predicted using miRNA target prediction tools. Representative immunoblots and quantification of TXNIP in (B) primary cultured hepatocytes transfected with NC, miR-144-3p, or miR-486a-3p mimics (*n* = 4) and (C) the liver (*n* = 7). (D) *Txnip and Ccna2* mRNA levels of primary cultured hepatocytes treated with EVs or vehicle (PBS) (*n* = 6). (E) Dual-luciferase assay in Hepa 1–6 cells transfected with 0.2 μg of the 3’-UTR reporting vector containing Firefly luciferase gene followed by *Txnip* 3’-UTR sequence (V) or co-transfected with 0.2 μg of the same vector and 100 nM of negative control (NC), miR-144-3p, or miR-486a-3p mimics (*n* = 6). (F) *Txnip*, *Ccna2*, and *Mki67* mRNA levels of primary cultured hepatocytes transfected with 10 nM of scrambled (NC) or *Txnip*-targeting siRNA (siTxnip_1) (*n* = 4). *p < 0.05, **p < 0.01, ***p < 0.001, ****p < 0.0001.

To elucidate the molecular mechanism by which miR-144-3p and miR-486a-3p promote hepatocyte proliferation, we screened the candidate target genes of these miRNAs by bioinformatics analyses. Using miRNA target prediction tools TargetScan [26] and RNAhybrid [27], we found that *Txnip* (thioredoxin-interacting protein) can be a common target gene of both miR-144-3p and miR-486a-3p (Fig 5A). TXNIP is a member of the α-arrestin family and has been identified as a protein that regulates the cellular redox homeostasis [28]. Recent studies have demonstrated that TXNIP also plays an inhibitory role in cellular growth, and one of its mechanisms is to act suppressively on the *Ccna2* promoter [29].

We conducted Western blotting for TXNIP in primary cultured hepatocytes transfected with miRNA mimics and found that the TXNIP levels were actually decreased in both hepatocytes transfected with miR-144-3p or miR-486a-3p mimics compared with the negative control miRNA mimics (Fig 5B). It is noteworthy that the TXNIP expression was also significantly reduced in the liver of iFIRKO mice (Fig 5C). Furthermore, primary cultured hepatocytes treated with serum EVs of iFIRKO mice exhibited higher expression of *Ccna2* with lower expression of *Txnip* than those treated with serum EVs of control mice (Fig 5D).

To determine whether *Txnip* is a direct target of miR-144-3p and miR-486a-3p, we next conducted dual-luciferase 3’-UTR reporter assays. Hepa 1–6 cells were co-transfected with miRNA mimics and expression vectors of the Firefly luciferase gene, followed by the *Txnip* 3’-UTR sequence. The activity levels of Firefly luciferase were significantly reduced in the vector-introduced cells with transfection of miR-144-3p or miR-486a-3p mimics compared with those with transfection of negative control miRNA mimics (Fig 5E).

Finally, we investigated whether the suppression of *Txnip* expression alone is sufficient to promote hepatocyte proliferation. Primary cultured hepatocytes transfected with *Txnip*-targeting siRNAs exhibited higher levels of *Mki67* expression than those transfected with scrambled control siRNA, accompanied by an upregulated expression of *Ccna2* (Fig 5F, S5 Fig).

These observations indicate that *Txnip* is a direct target of miR-144-3p and miR-486a-3p and that the suppression of *Txnip* gene expression by these miRNAs would be responsible for the hepatocyte proliferation in the liver of iFIRKO mice.

## Discussion

Regenerative medicine is expected to be a novel therapeutic strategy to overcome diseases that remain incurable until now. MSCs are promising cells for the regeneration of damaged tissues owing to their anti-apoptotic, cell proliferative, and immunoregulatory properties [19]. Liver cirrhosis is a life-threatening condition with decreased viable hepatocytes, and since there is no effective treatment option other than liver transplantation, regenerative therapy using MSCs has been vigorously investigated for this disease [22, 30]. While animal studies have achieved compelling results of MSC administration for liver regeneration, the efficacy observed in current clinical studies appears to be modest and lower than expected [22]. The reasons for this are still unknown, and to achieve therapeutic applications, it seems necessary to identify the molecules responsible for the regenerative effects and to elucidate the precise mechanism.

Recently, EVs have been recognized to play significant roles in tissue regeneration as the administration of EVs secreted from MSCs instead of MSCs themselves has shown similar effects in animal models of liver fibrosis [31]. Since identifying and administering the responsible molecule in EVs is the most reliable and safest way to realize the desired effect, many researchers are searching for it in the contents of EVs secreted from cultured MSCs. However, few definitive molecules have been found to exert a regenerative effect on liver cirrhosis to date, and it is assumed that the profile of EVs secreted from MSCs cultured and expanded on plastic dishes differs from that of EVs actually secreted from MSCs *in vivo* [32].

Adipose tissue has relatively abundant MSCs as a subset of ASPCs and is recognized as a source of MSCs for regenerative therapy. In this study, we used a unique mouse model in which ASPCs are acutely increased in the adipose tissue and examined serum EV-miRNAs that are presumably influenced by alterations in their release from the adipose tissue. We found increased hepatocyte proliferation in the liver of iFIRKO mice and that the serum EVs of iFIRKO mice are capable of promoting cell proliferation in primary cultured hepatocytes independent of insulin action. We also found that miR-144-3p and miR-486a-3p were simultaneously increased in the serum EVs and liver of iFIRKO mice and demonstrated that miR-144-3p and miR-486a-3p both promote hepatocyte proliferation by suppressing *Txnip* expression. One of the limitations of this study is that quantitative comparison of miRNA in each organ relies on the comparative CT method, the accuracy of which is reportedly not as high as other methods [33]. Also, elevated levels of miRNA in the liver do not necessary indicate that these miRNAs are delivered directly from ASPCs increased in the adipose tissue to the hepatocytes via circulating EVs. However, the fact that primary forms of these miRNAs were upregulated in the adipose tissue but not in the liver in mice with adipocyte-specific acute insulin receptor knockout would support the delivery of miRNA from adipose tissue to the liver.

TXNIP was originally identified in HL-60 cells as a protein upregulated by vitamin D3 treatment. At present, it is known to have diverse functions in inflammation and cell proliferation [34, 35]. A previous study using cell lines reported that TXNIP inhibits *Ccna2* promoter activity and induces cell cycle arrest [29], and we confirmed that *Txnip* knockdown using siRNA upregulates *Ccna2* expression in primary cultured hepatocytes in this study. *Txnip* expression was decreased in hepatocytes transfected with miR-144-3p or miR-486a-3p, in hepatocytes treated with serum EVs of iFIRKO mice, and in the liver of iFIRKO mice, all accompanied by increased *Ccna2* and *Mki67* expressions. This suggests that *Txnip* suppression by these miRNAs play an important role in hepatocyte proliferation. Improved recovery from liver injury by enhancing hepatocyte proliferation has been reported in mice [36], and previous research demonstrated that *Txnip* knockout mice show accelerated liver generation following partial hepatectomy [37]. Therefore, miR-144-3p and miR-486a-3p might have therapeutic value against acute liver failure and liver cirrhosis with reduced endogenous regenerative capacity.

With the increase in the prevalence of obesity, an increasing number of patients are affected by NASH, one of the most common causes of liver cirrhosis nowadays [38]. It was reported that *Txnip* expression is elevated in the liver of high-fat diet-induced obese mice and that the levels of *Txnip* expression are positively related to the progression of NASH [39]. Because *Txnip* has been reported to activate NOD-like receptor family protein 3 (NLRP3) under oxidative stress [40], *Txnip* may be involved in exacerbating inflammation in addition to inhibiting regeneration in the liver of patients with NASH; in any case, *Txnip* suppression would improve the pathophysiology of NASH.

In general, the promotion of cell proliferation is associated with carcinogenesis, and reduced *TXNIP* expression has been reported in several cancers, including hepatocellular carcinoma [41]. It was also demonstrated that congenital *Txnip*-deficient mice exhibit a high incidence of hepatocellular carcinoma, with up to 66% of mice developing liver cancers by 20−24 months of age [42]. Nevertheless, transient suppression of *Txnip* by miRNAs may not result in such a high risk of carcinogenesis. Further studies are needed to determine the efficacy and safety of suppressing *Txnip* as a therapeutic target for incurable liver diseases including liver cirrhosis caused by NASH.

In conclusion, we provide evidence that several miRNAs are increased in serum EVs during acute adipose tissue regeneration with increased ASPCs. Among them were miR-144-3p and miR-486a-3p, which promote hepatocyte proliferation. Our study indicates that examining EV-miRNAs secreted from MSCs *in vivo* may lead to the discovery of previously unidentified molecules involved in regenerative therapy using MSCs and their EVs.

## Supporting information

S1 Fig iFIRKO mice exhibit severe lipodystrophy with insulin resistance. (A) Body weight, and (B) weights of inguinal WAT, epididymal WAT, and BAT. Levels of (C) blood glucose and (D) serum insulin. (E) *Insr* mRNA levels of the adipose tissue and liver. Representative images of HE staining of (F) the epididymal WAT and (G) the BAT. Original magnification, ×200. Scale bars, 100 μm. ***p < 0.001, ****p < 0.0001. *n* = 7.
(PDF)

S2 Fig Validation of increased serum EV-miRNAs by quantitative RT-PCR using spike-in control. Levels of serum EV-miRNAs normalized to those of spike-in control cel-miR-39-3p. **p < 0.01, ***p < 0.001, ****p < 0.0001. *n* = 10.
(PDF)

S3 Fig Levels of primary and mature form of miRNAs in the epididymal WAT and BAT of iFIRKO mice. Levels of (A) primary form and (B) mature form of miR-144-3p and miR-486a-3p in the epididymal WAT and the BAT. *p < 0.05, ****p < 0.0001. *n* = 7.
(PDF)

S4 Fig Primary cultured hepatocytes transfected with miR-144-3p or miR-486a-3p mimics exhibit cell proliferation. Representative images of Ki-67 (green) immunostaining of primary hepatocytes transfected with 10 nM of negative control (NC), miR-144-3p, or miR-486a-3p mimics. Blue: DAPI. Original magnification, ×200. Scale bars, 100 μm. *n* = 4.
(PDF)

S5 Fig Knockdown of *Txnip* in primary cultured hepatocytes using another siRNA. *Txnip*, *Ccna2*, and *Mki67* mRNA levels of primary cultured hepatocytes transfected with 10 nM of scrambled or *Txnip*-targeting siRNA (siTxnip_2). *p < 0.05, ***p < 0.001. *n* = 3.
(PDF)

S1 Table List of primers. (PDF)

S1 Raw images All original blot images. (PDF)
