# Supplementary figures and images for: Increased serum extracellular vesicle miR-144-3p and miR-486a-3p in a mouse model of adipose tissue regeneration promote hepatocyte proliferation by targeting Txnip
Source: PLoS One. 2023 May 4;18(5):e0284989. doi: 10.1371/journal.pone.0284989 (PMC10159167; doi:10.1371/journal.pone.0284989)

**A**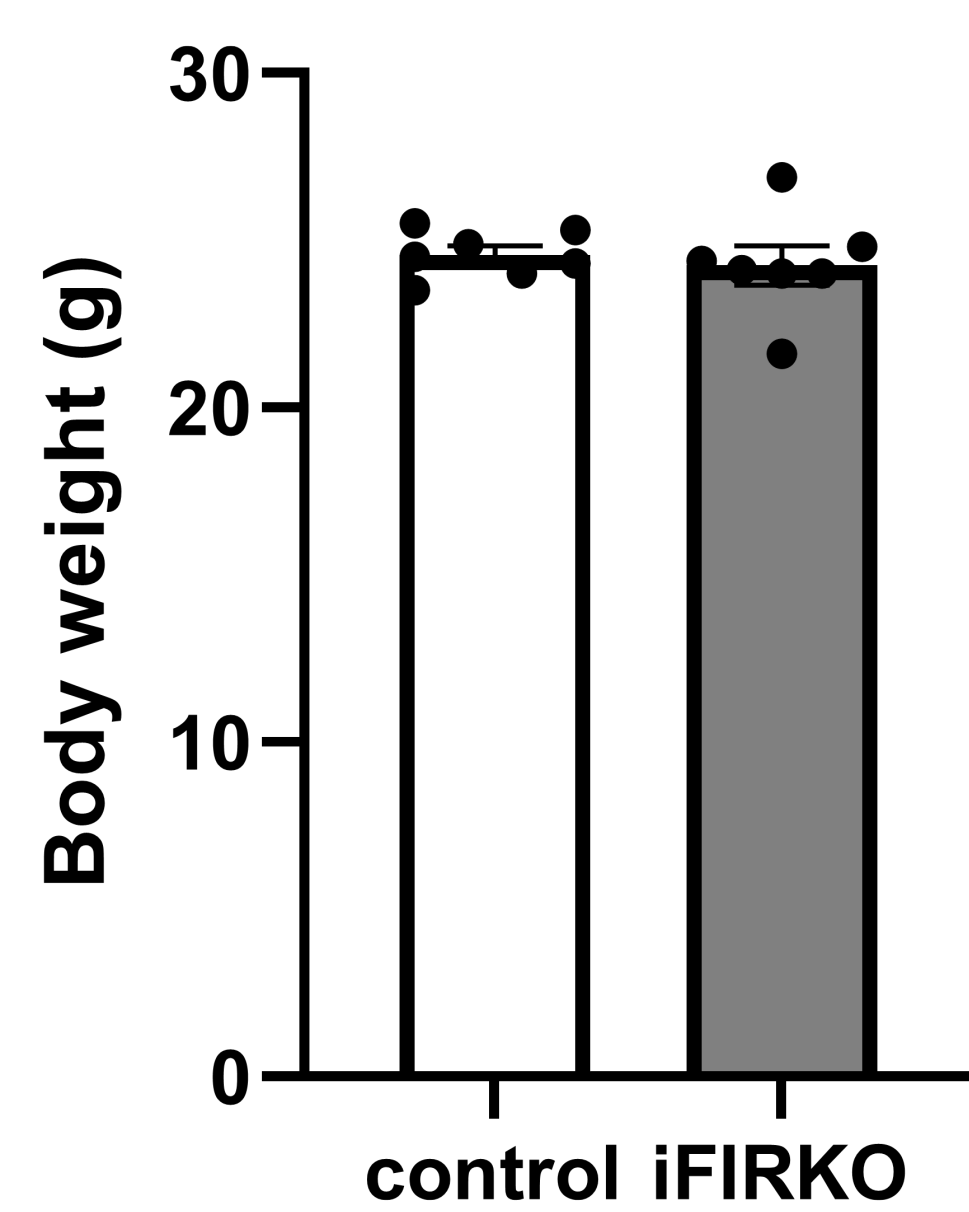**B**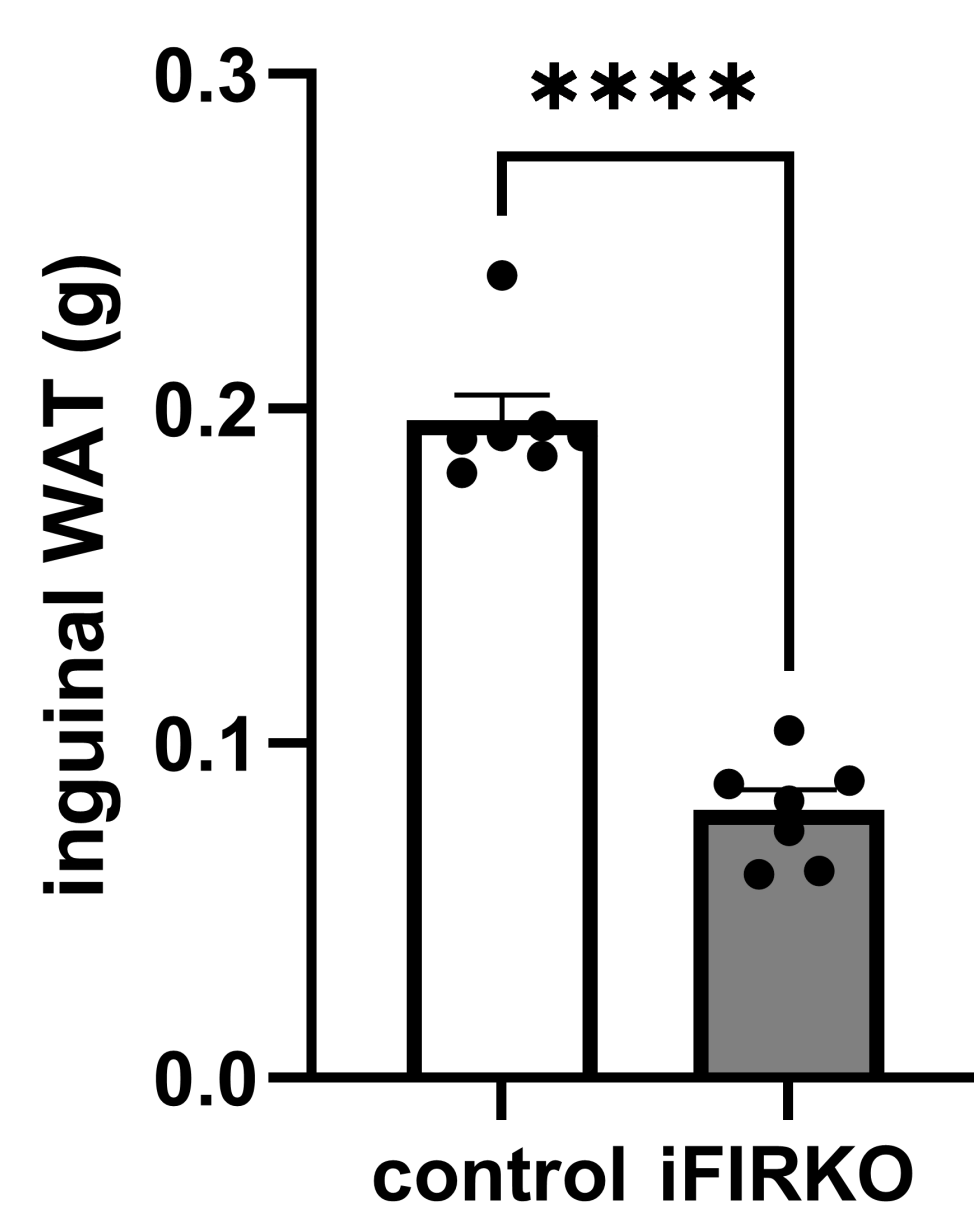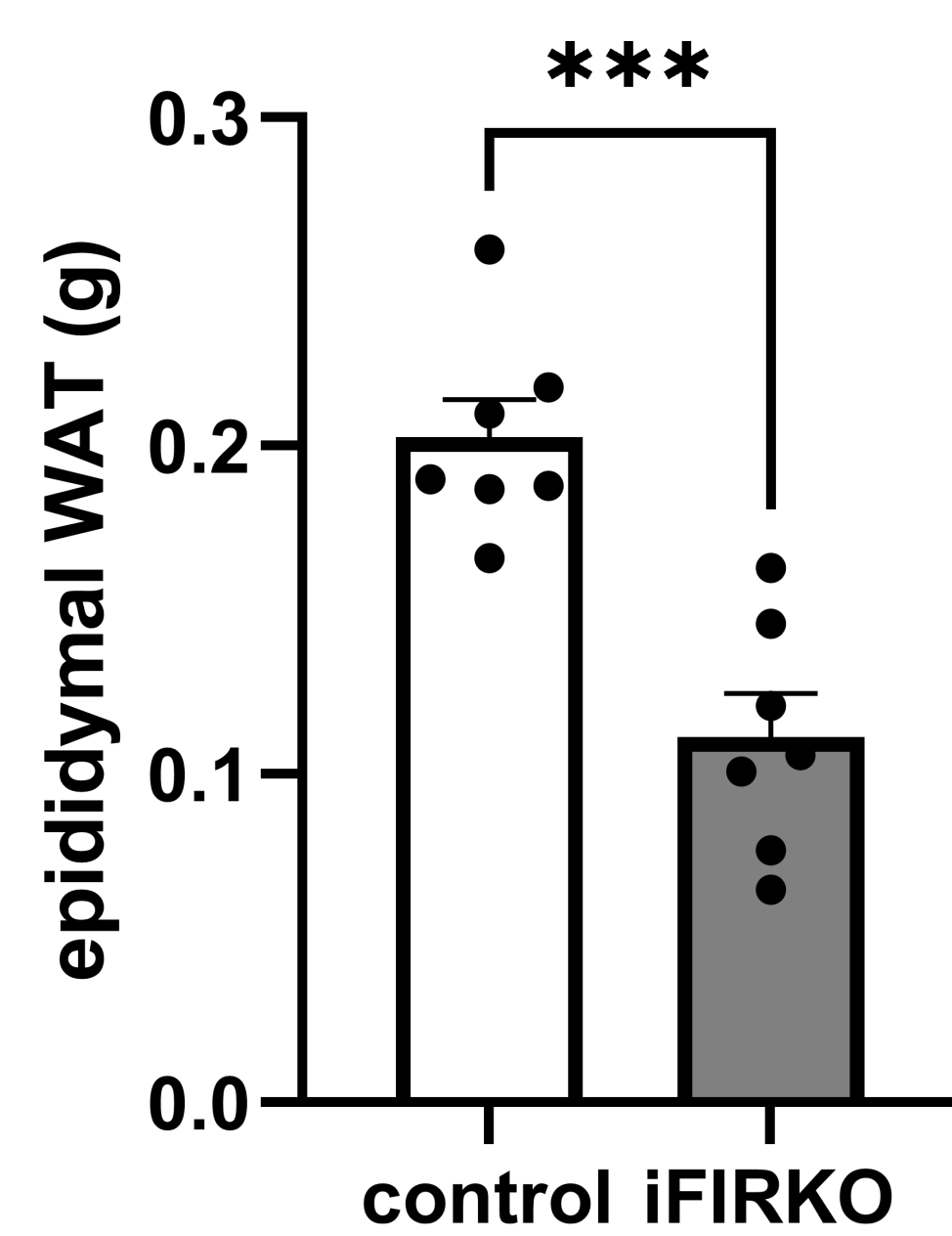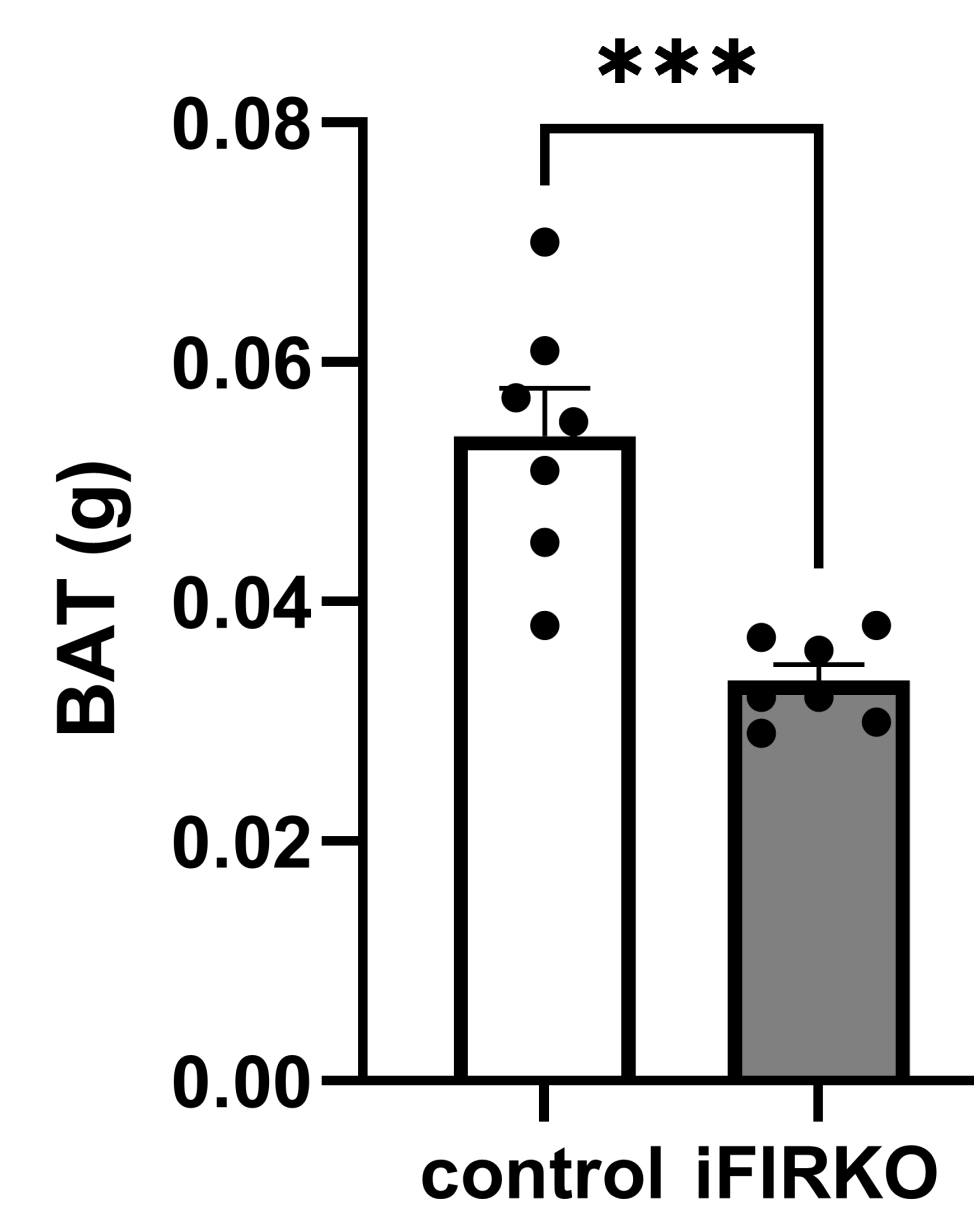**C**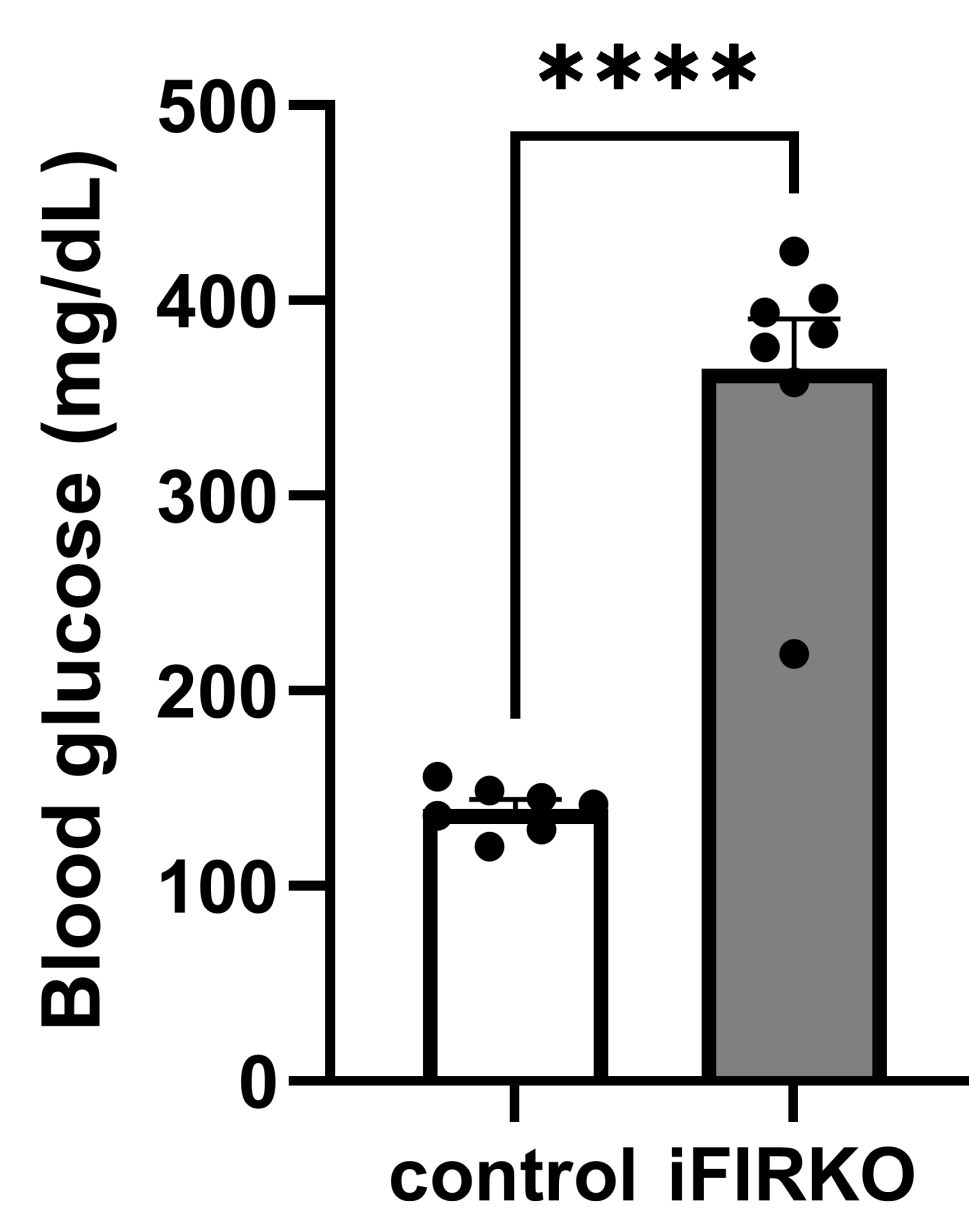**D**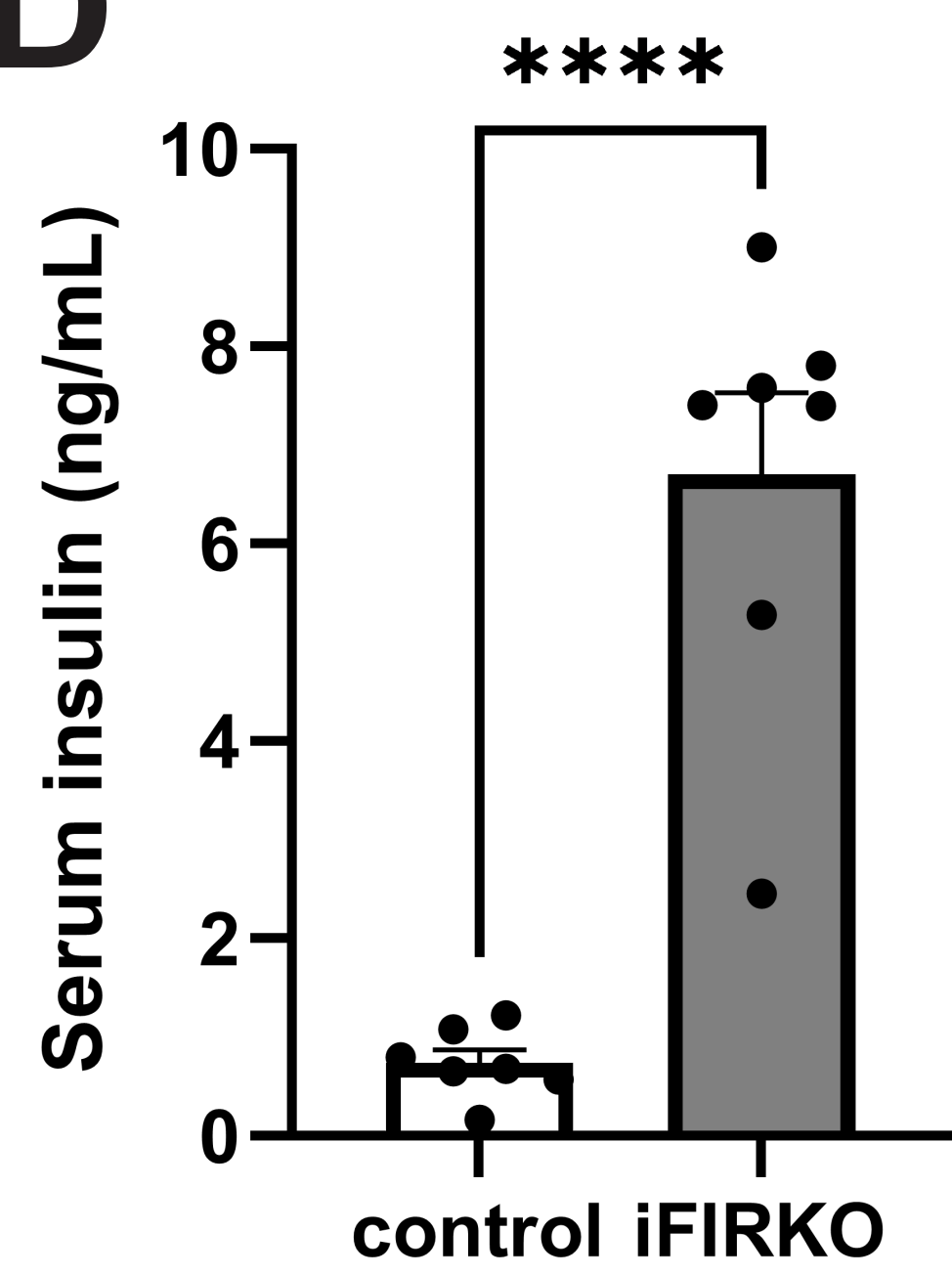**E**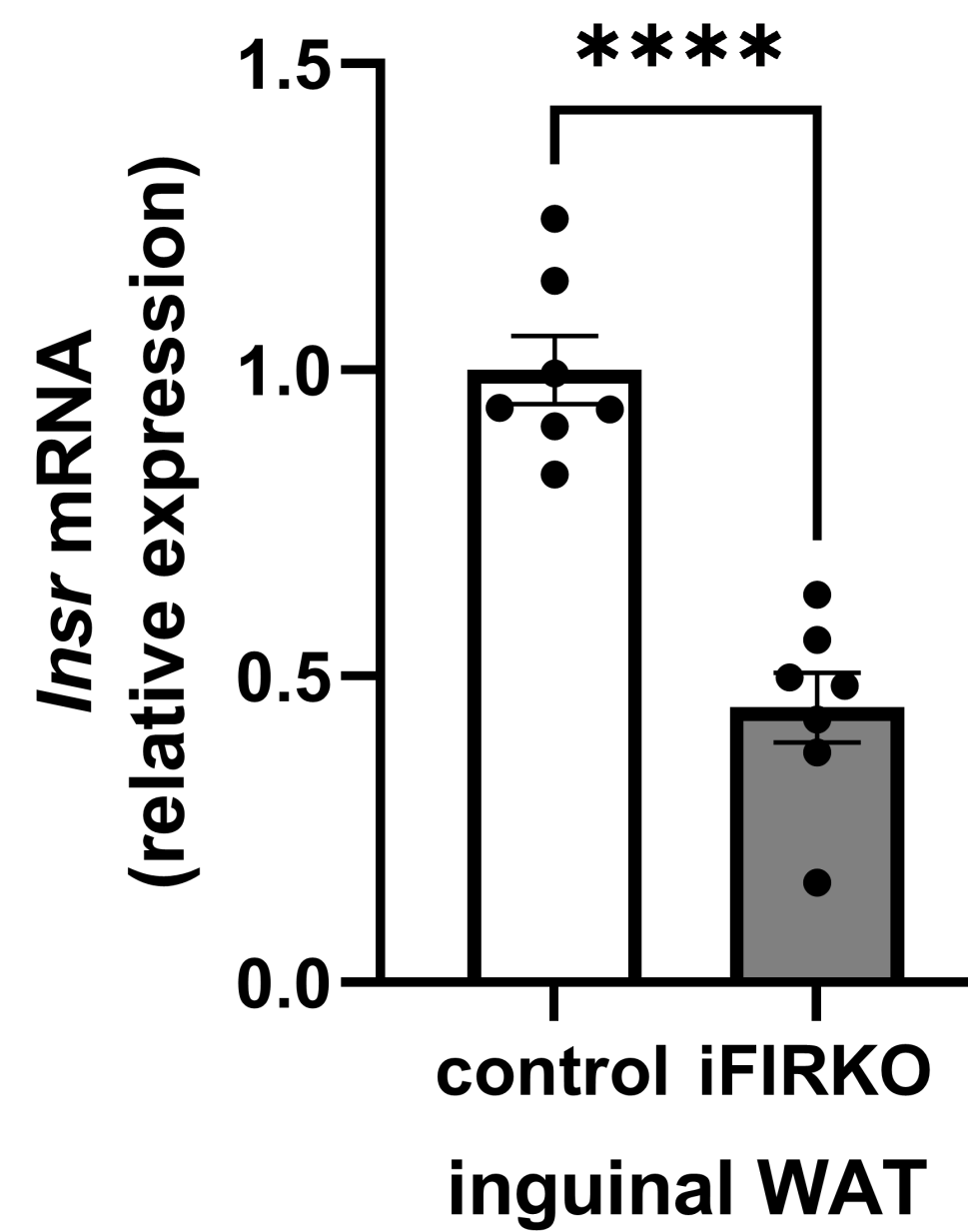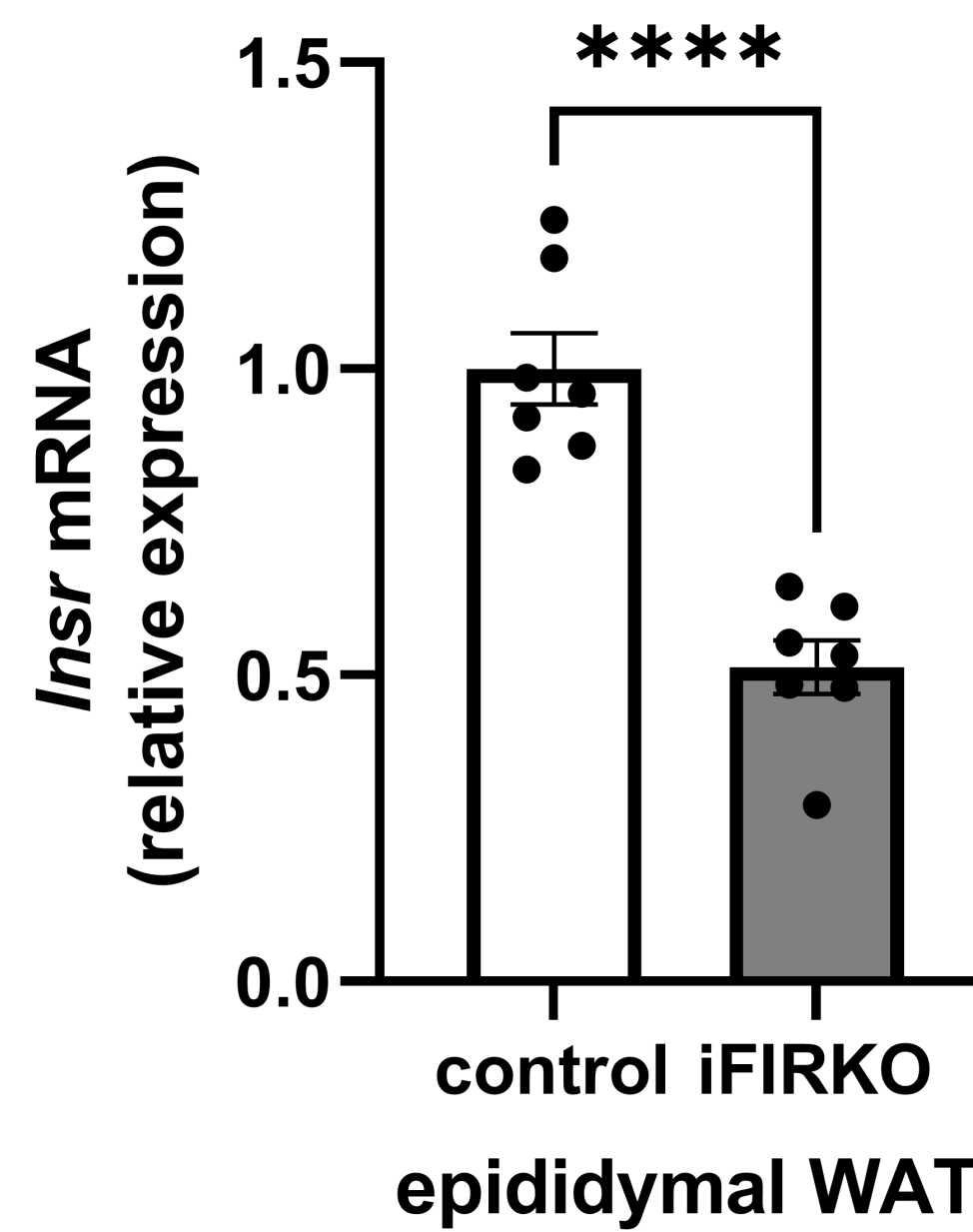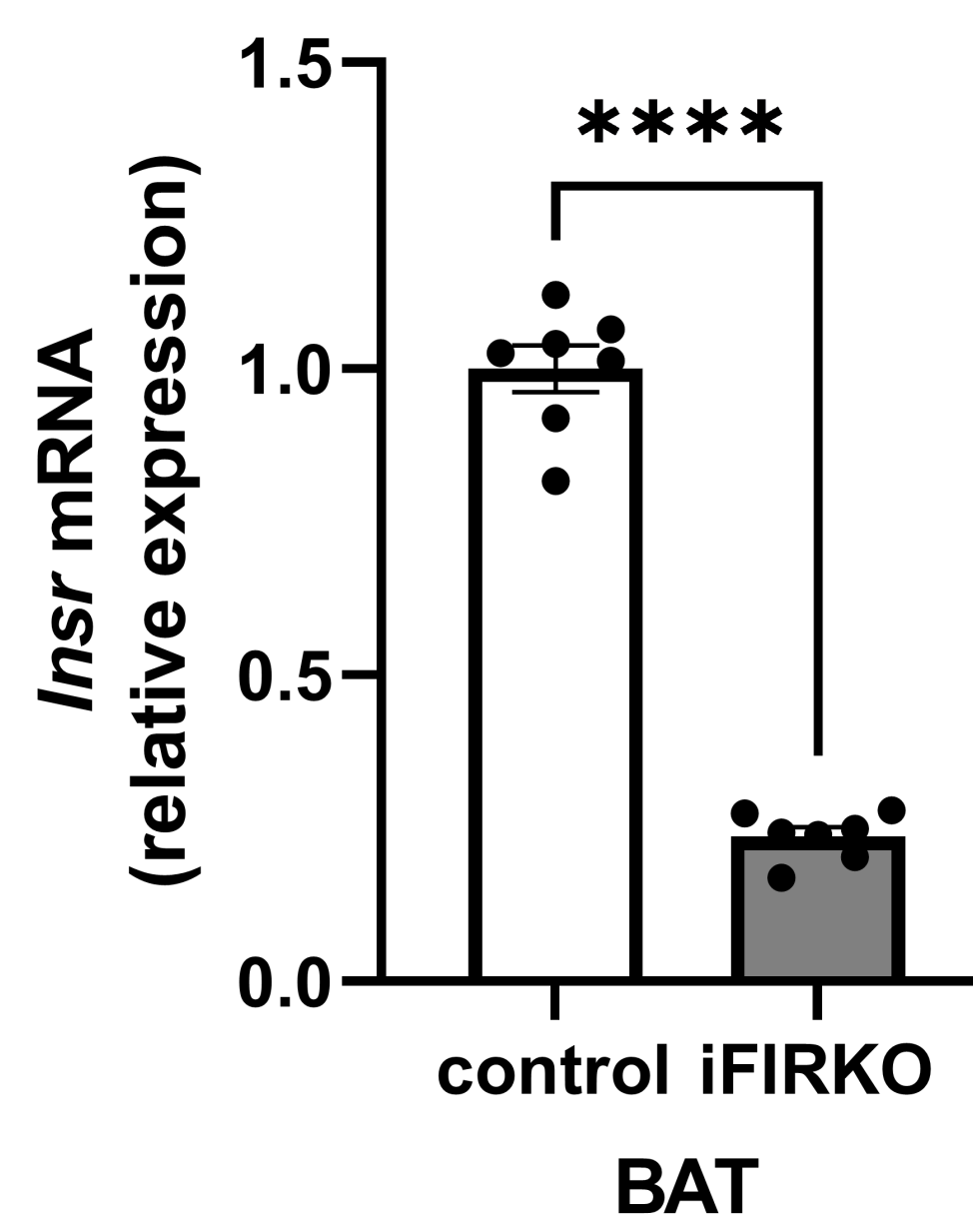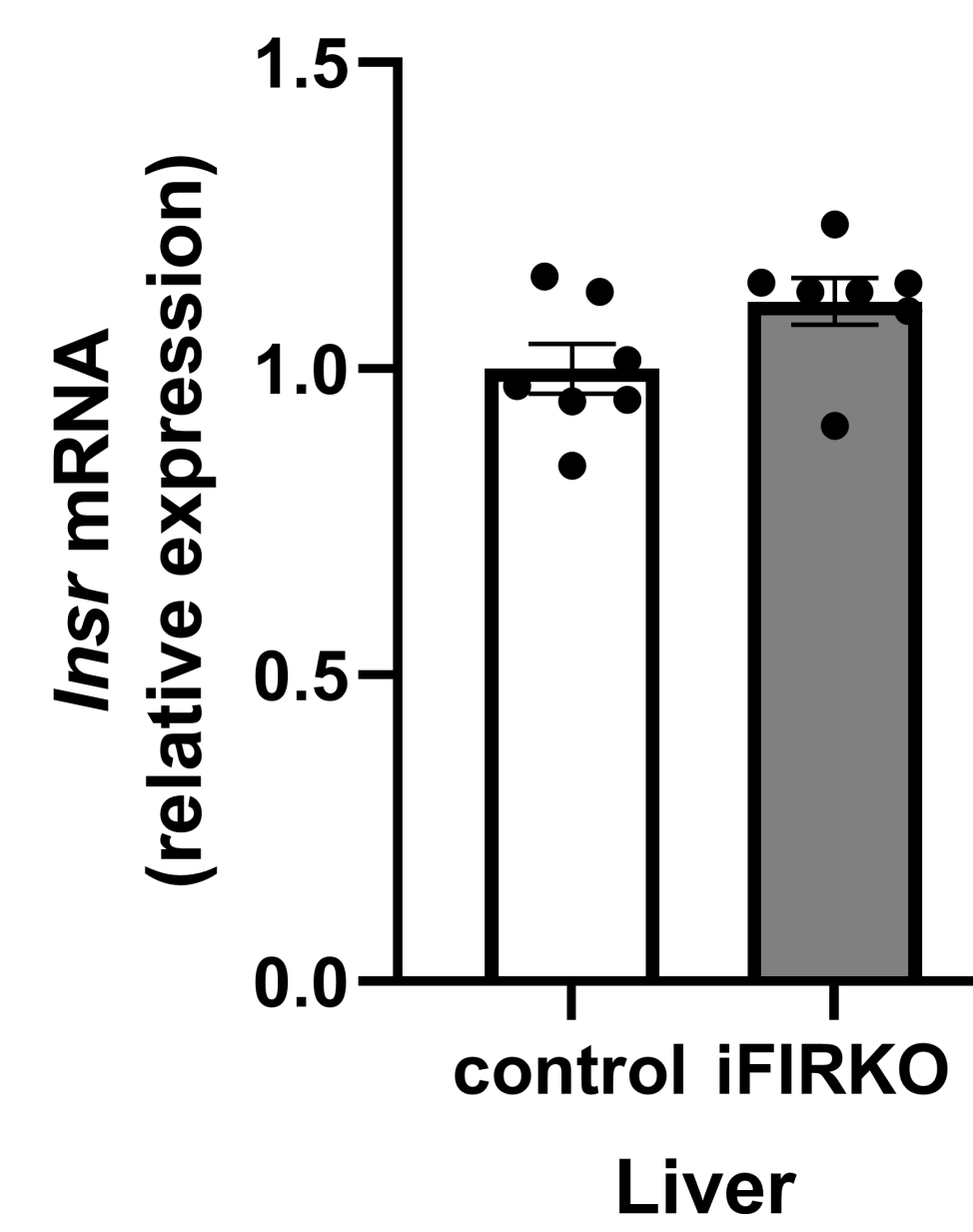**F**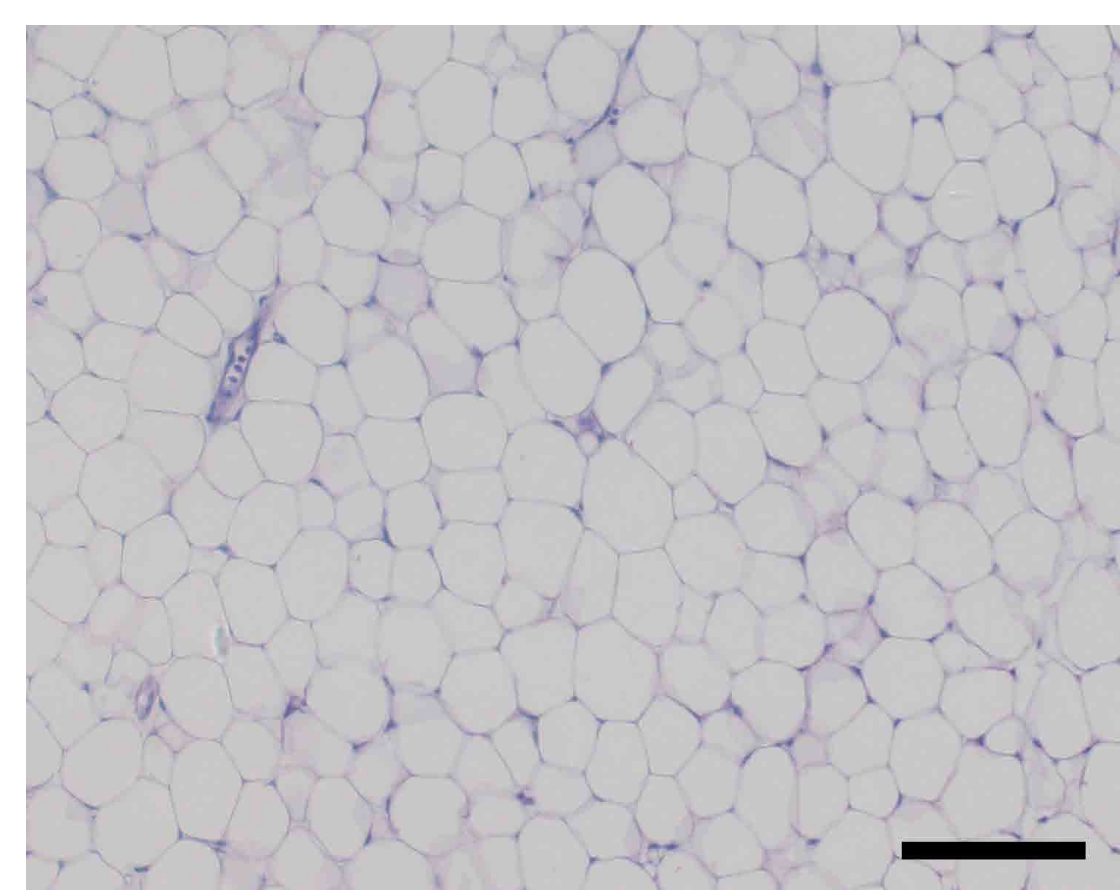

control

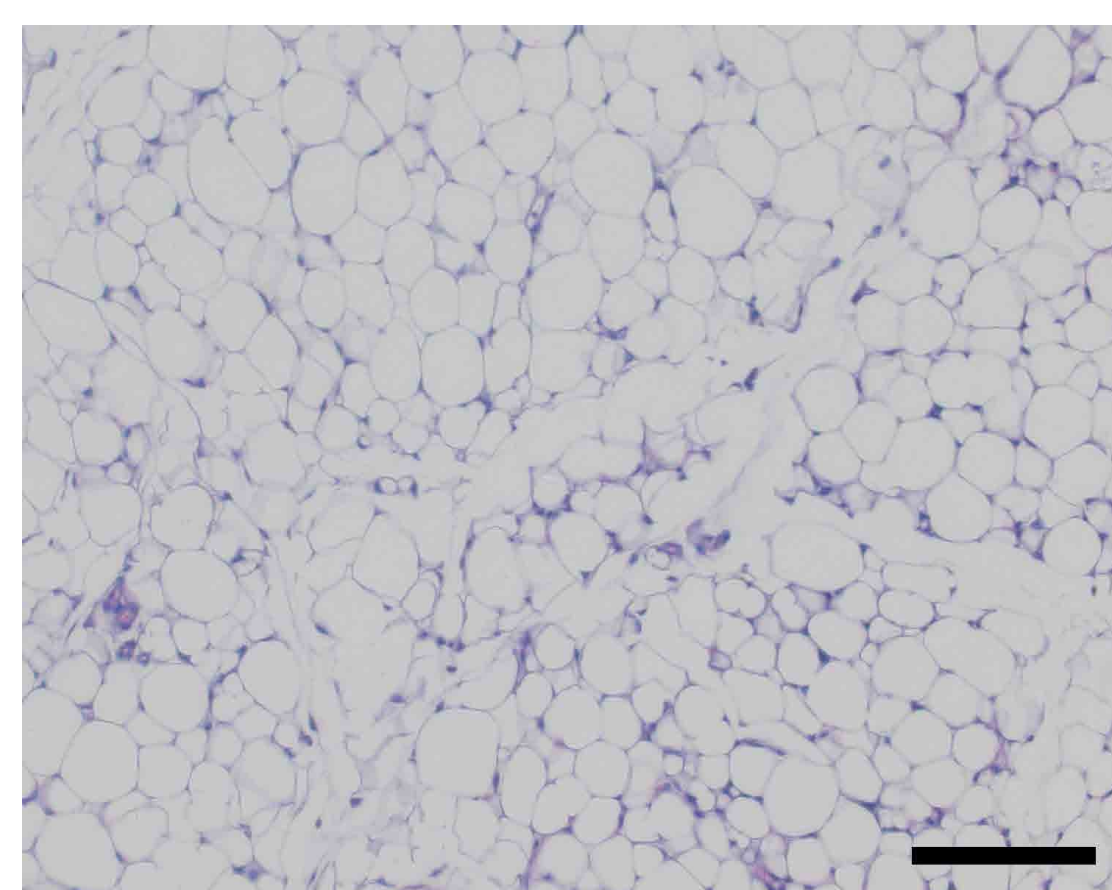

iFIRKO

**G**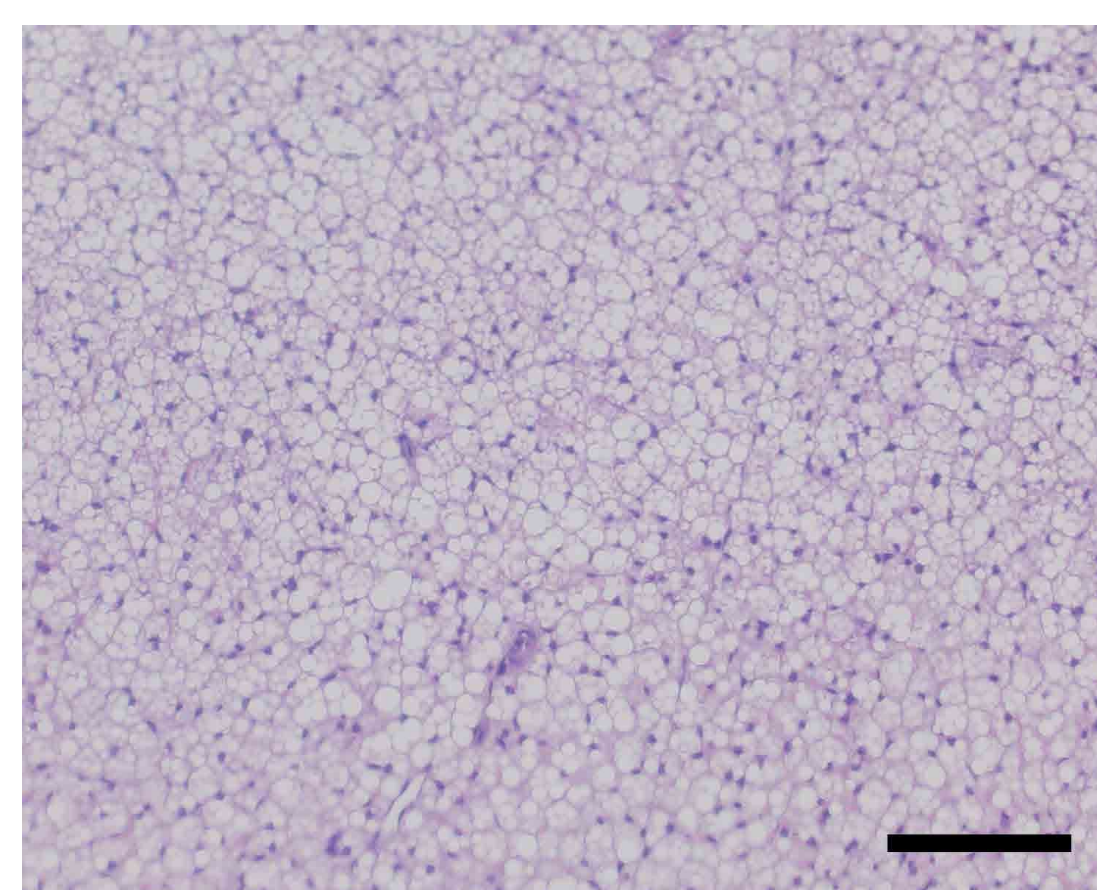

control

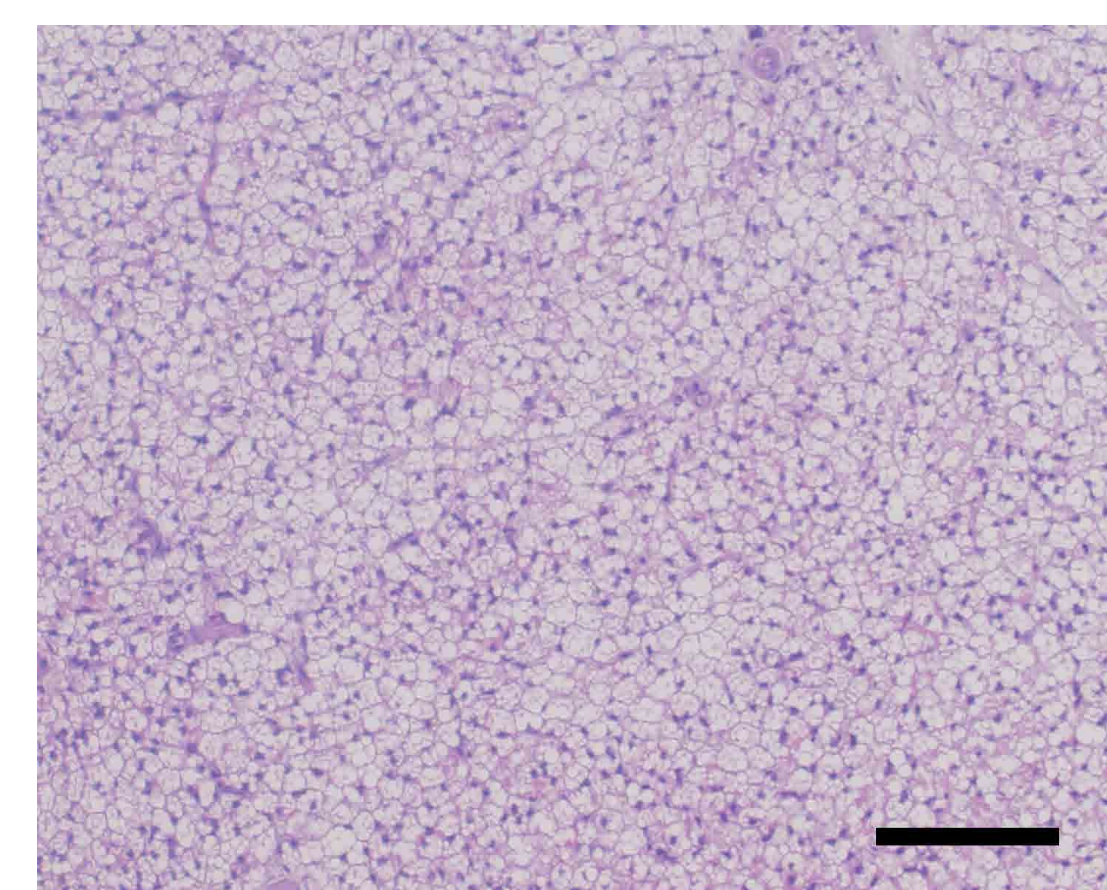

iFIRKO

Supplement: S1 Fig — (A) Body weight, and (B) weights of inguinal WAT, epididymal WAT, and BAT. Levels of (C) blood glucose and (D) serum insulin. (E) Insr mRNA levels of the adipose tissue and liver. Representative images of HE staining of (F) the epididymal WAT and (G) the BAT. Original magnification, ×200. Scale bars, 100 μm. ***p < 0.001, ****p < 0.0001. n = 7. (PDF) [file pone.0284989.s001.pdf]

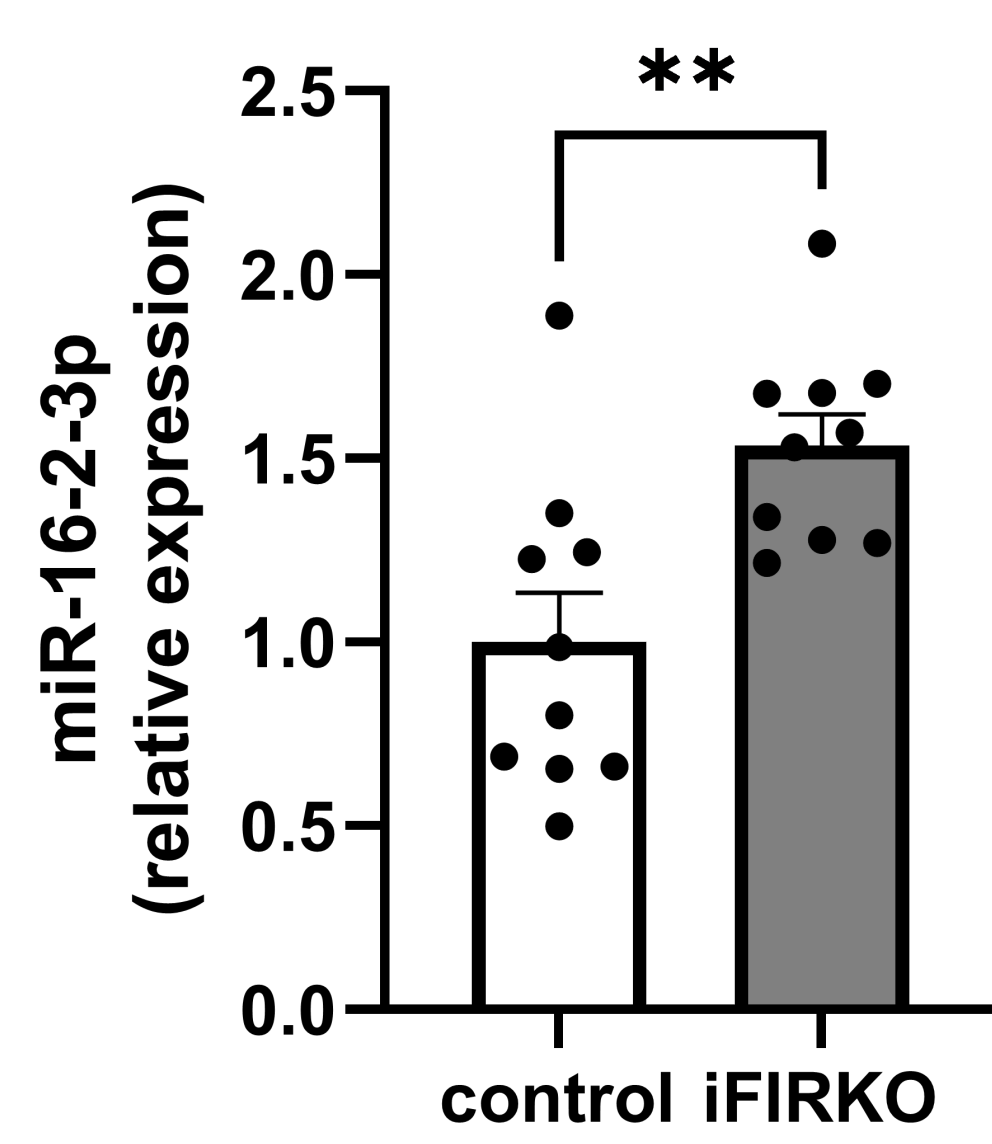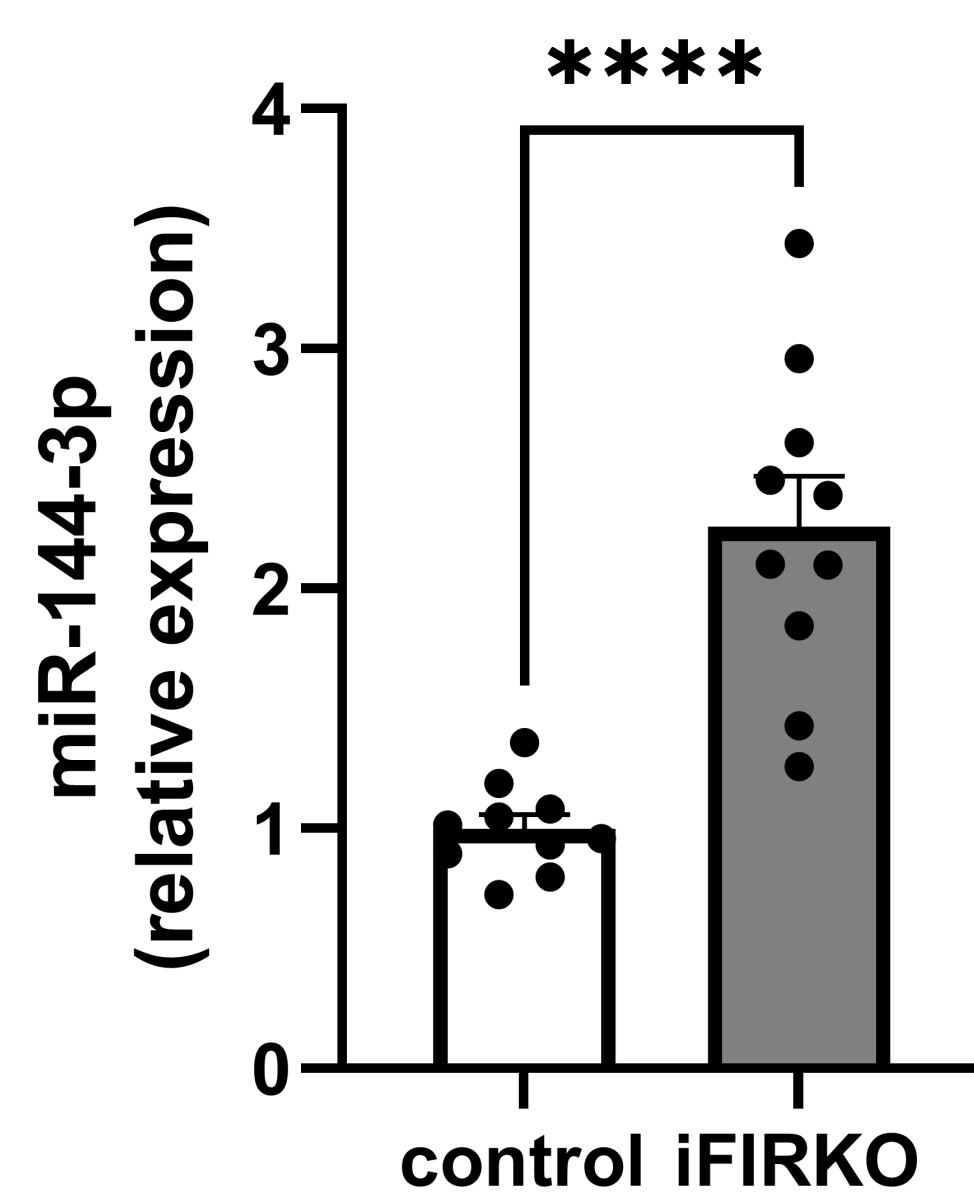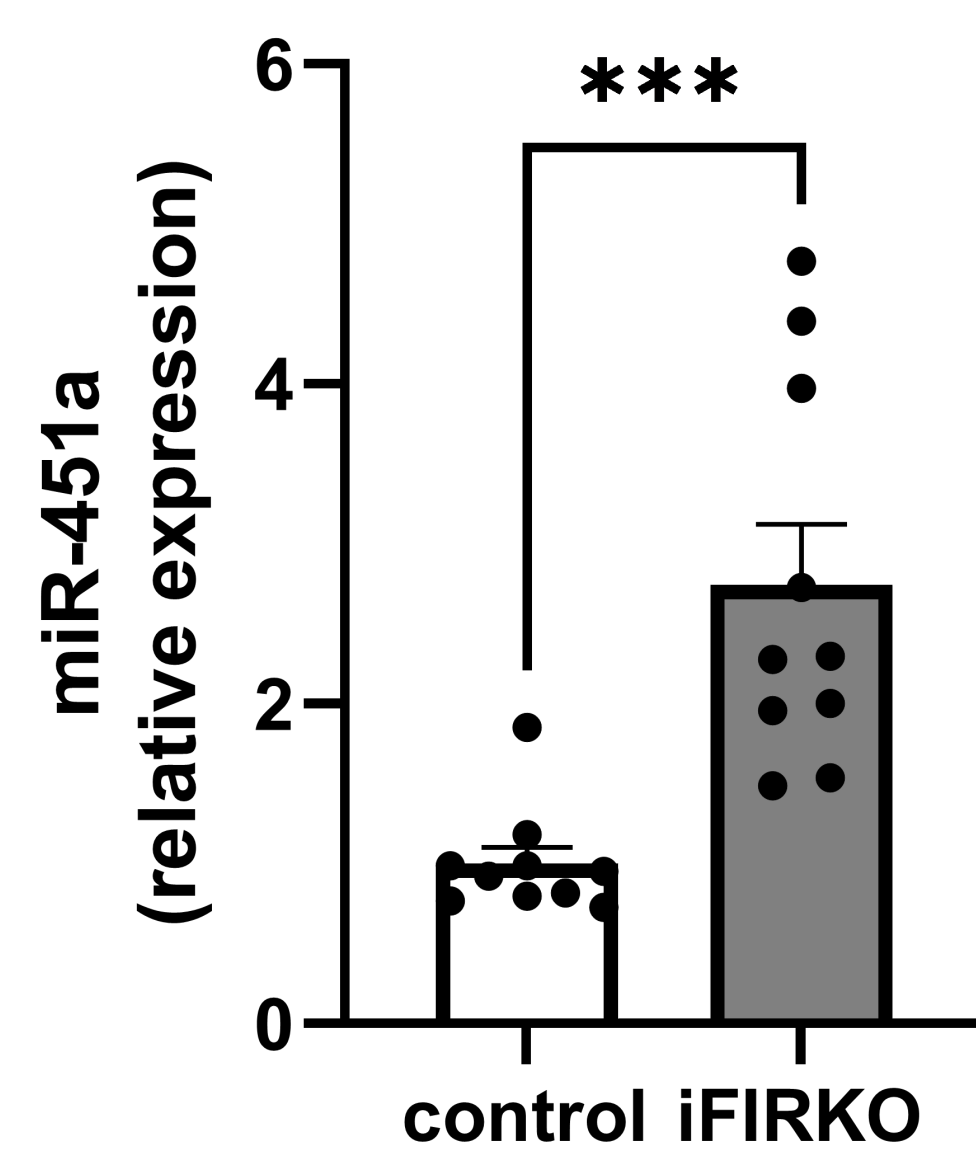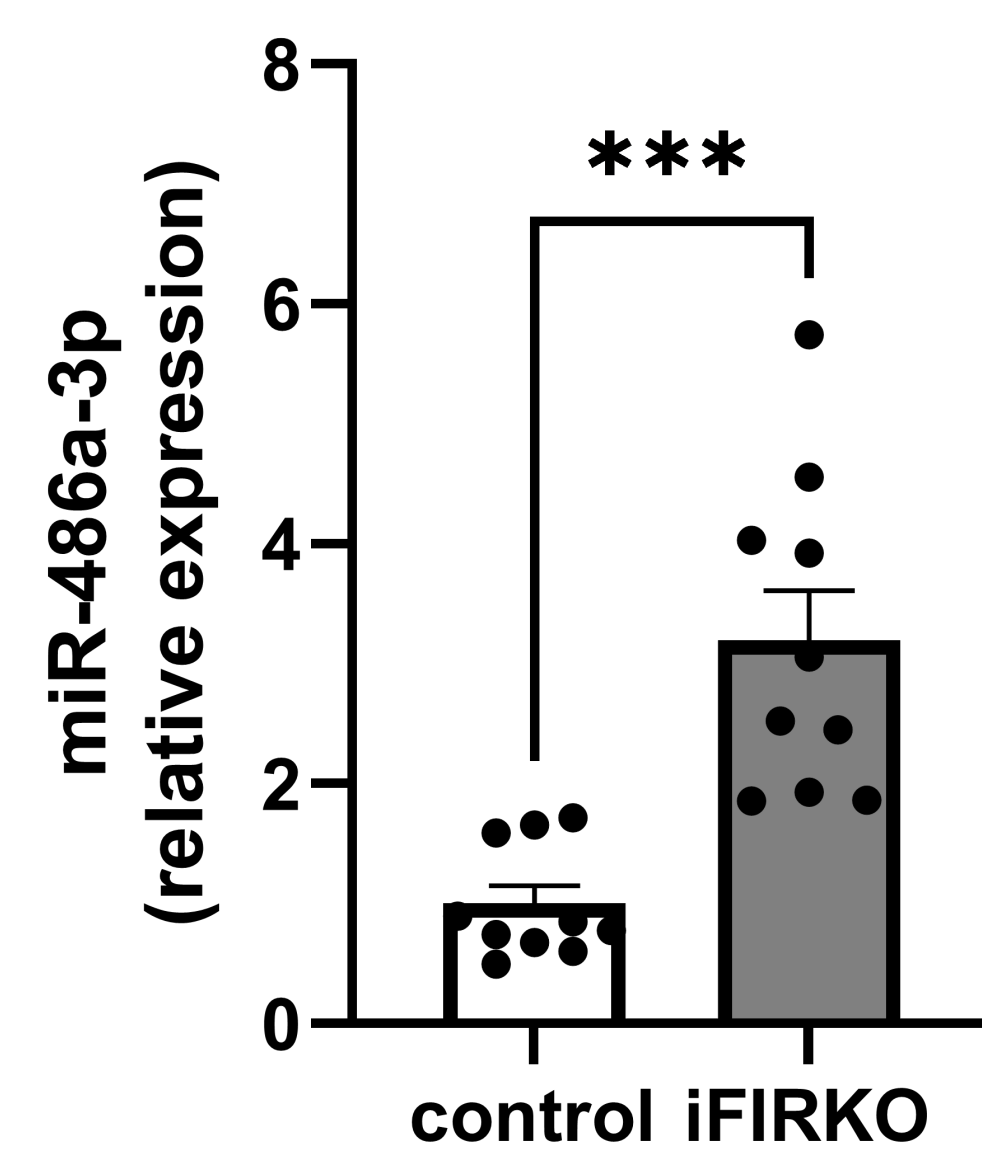

Supplement: S2 Fig — Levels of serum EV-miRNAs normalized to those of spike-in control cel-miR-39-3p. **p < 0.01, ***p < 0.001, ****p < 0.0001. n = 10. (PDF) [file pone.0284989.s002.pdf]

**A**

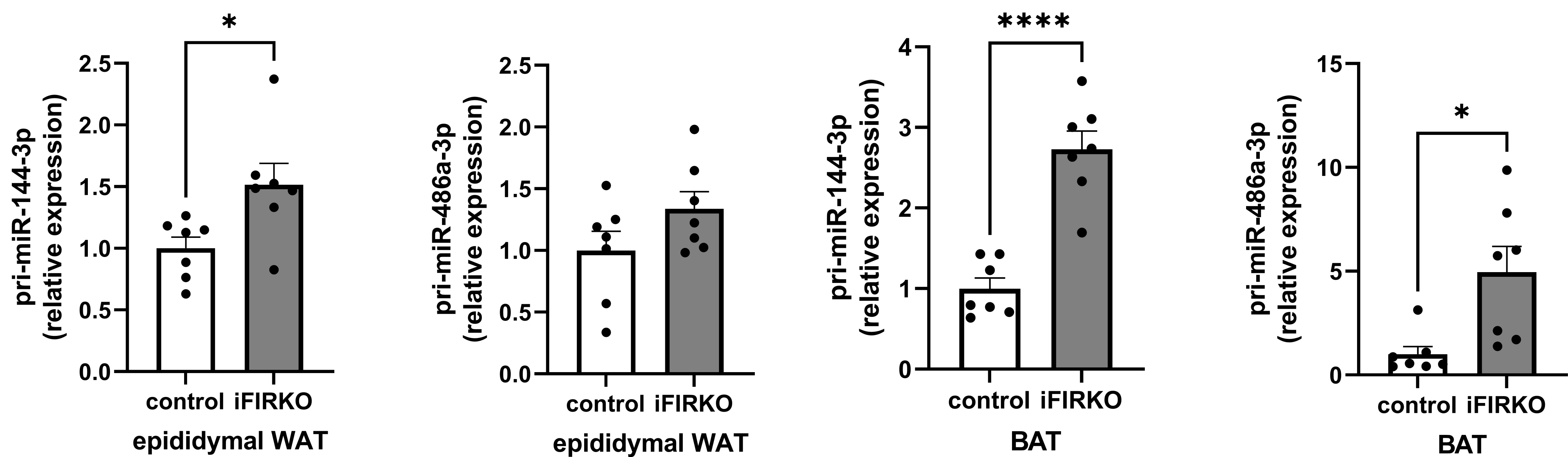

**B**

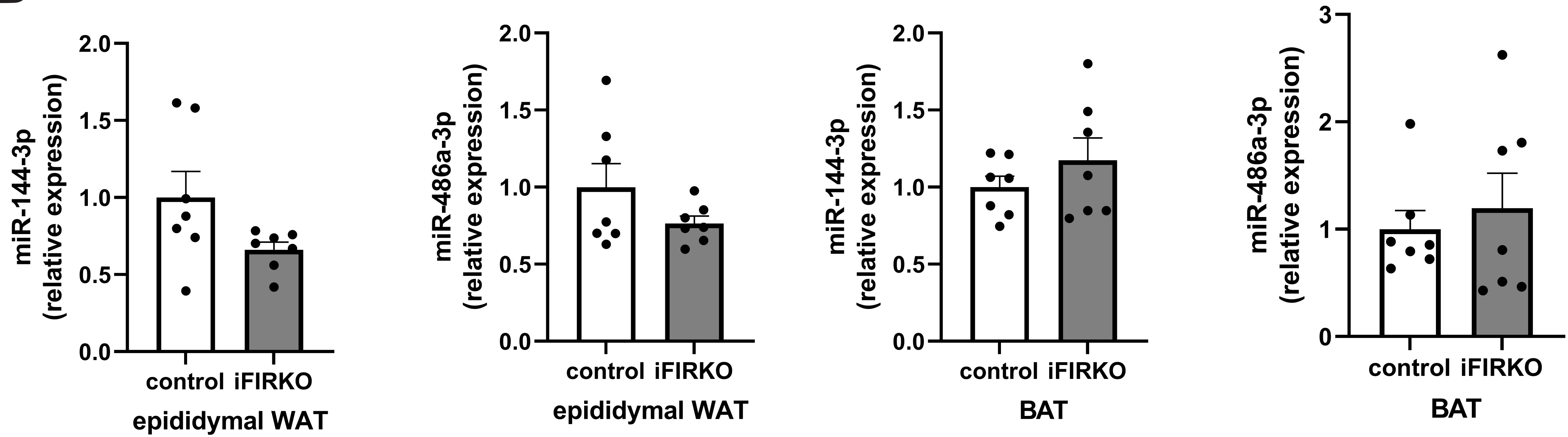

Supplement: S3 Fig — Levels of (A) primary form and (B) mature form of miR-144-3p and miR-486a-3p in the epididymal WAT and the BAT. *p < 0.05, ****p < 0.0001. n = 7. (PDF) [file pone.0284989.s003.pdf]

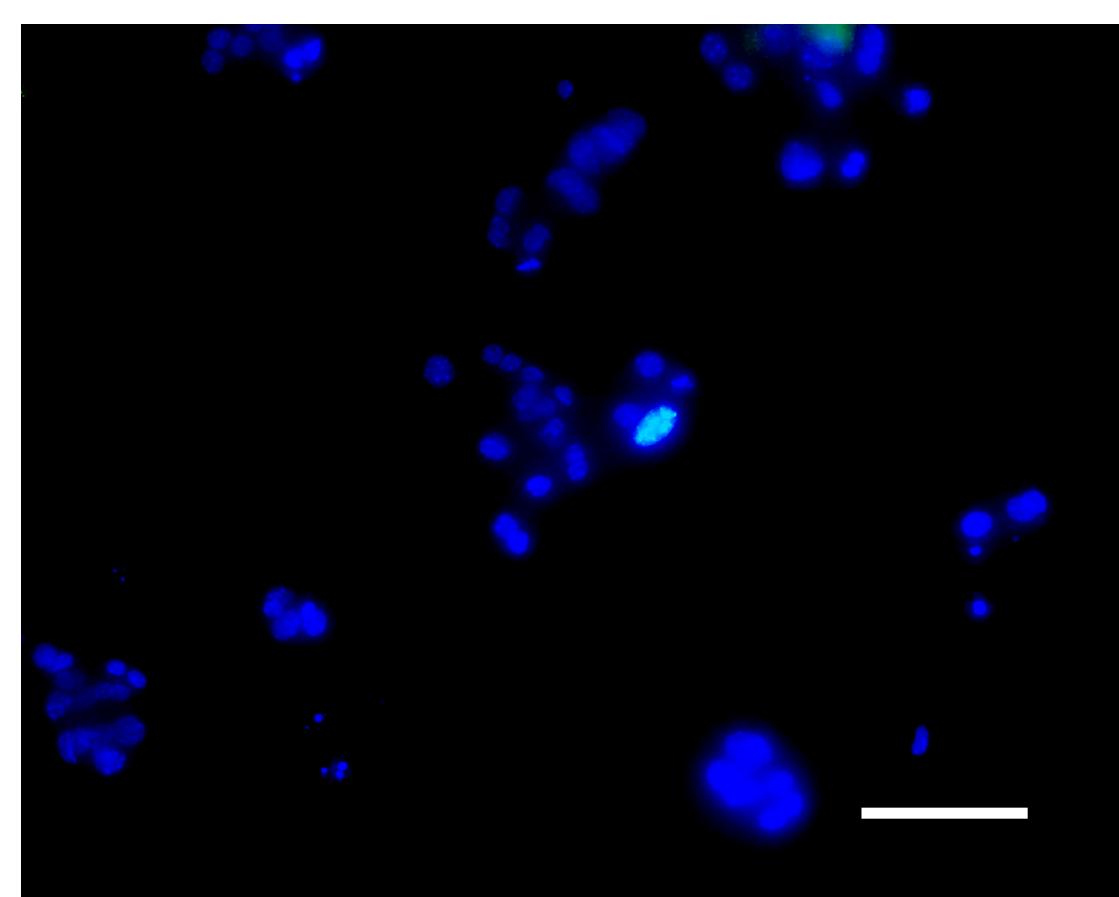

NC

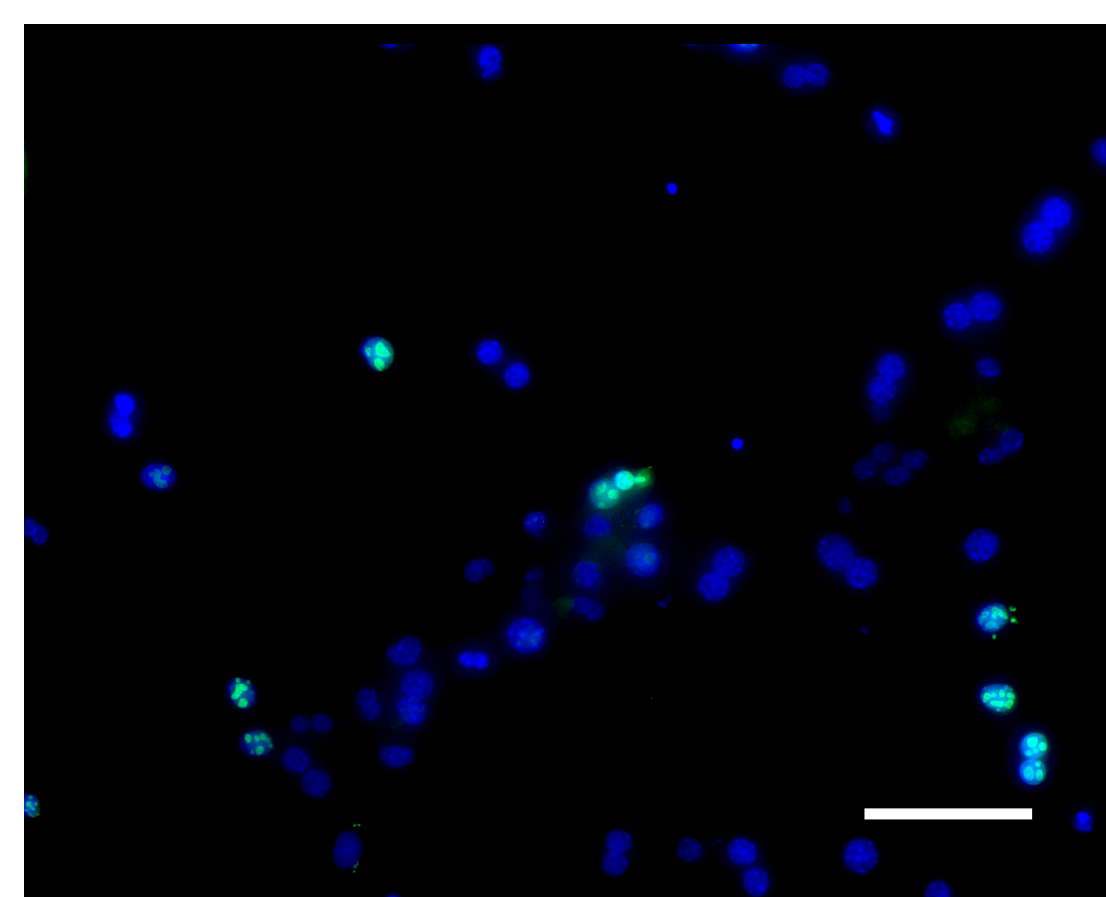

miR-144-3p

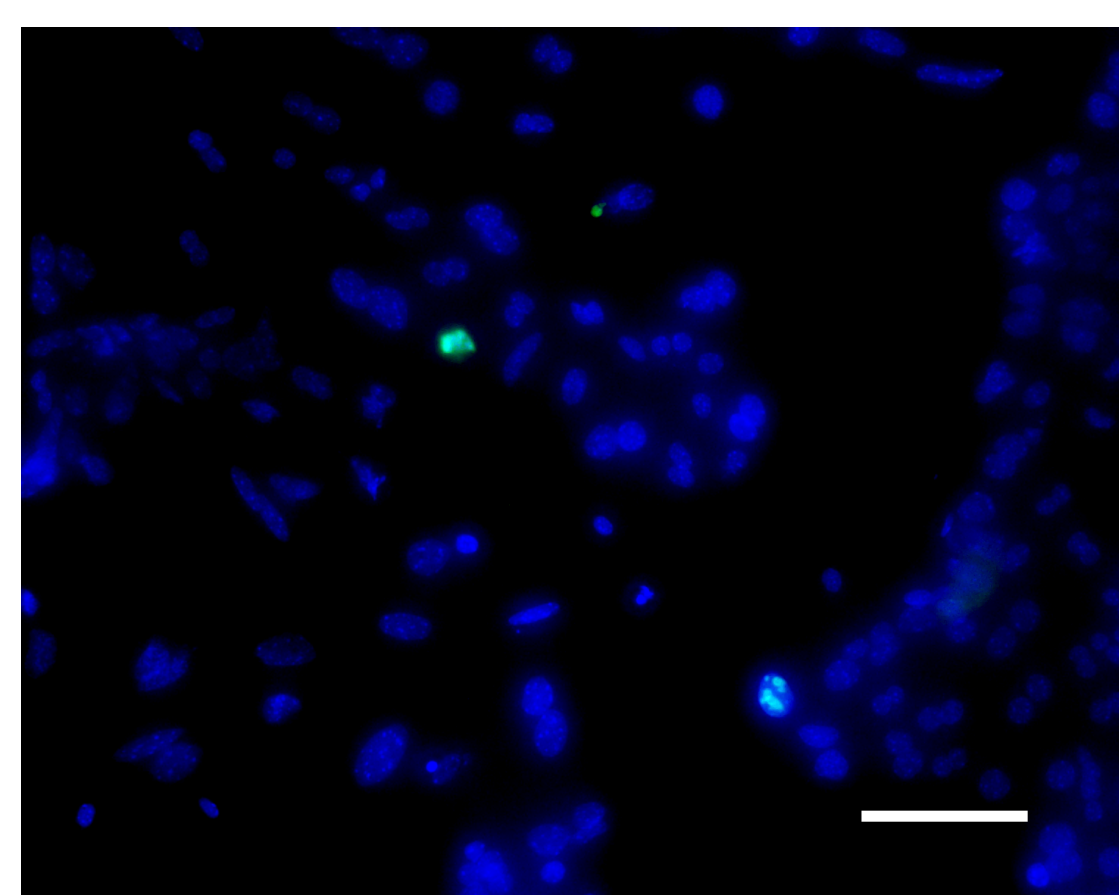

NC

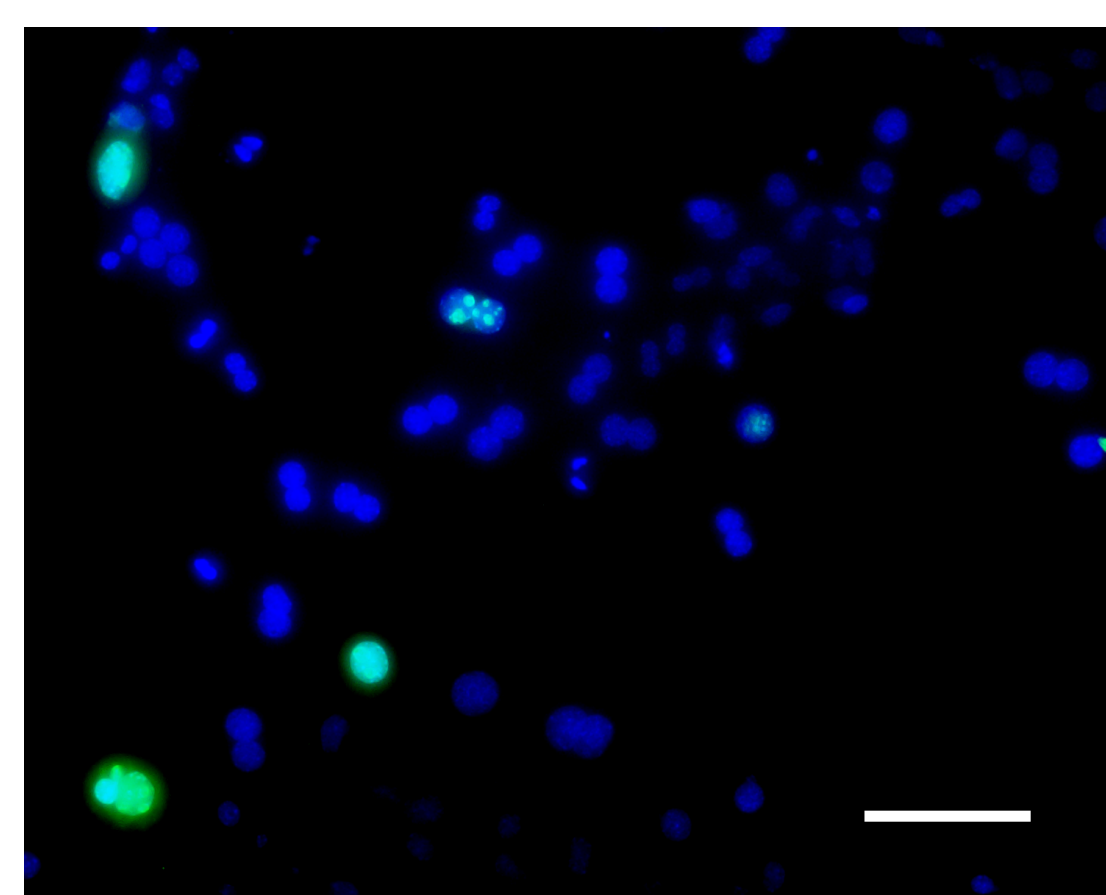

miR-486a-3

Supplement: S4 Fig — Representative images of Ki-67 (green) immunostaining of primary hepatocytes transfected with 10 nM of negative control (NC), miR-144-3p, or miR-486a-3p mimics. Blue: DAPI. Original magnification, ×200. Scale bars, 100 μm. n = 4. (PDF) [file pone.0284989.s004.pdf]

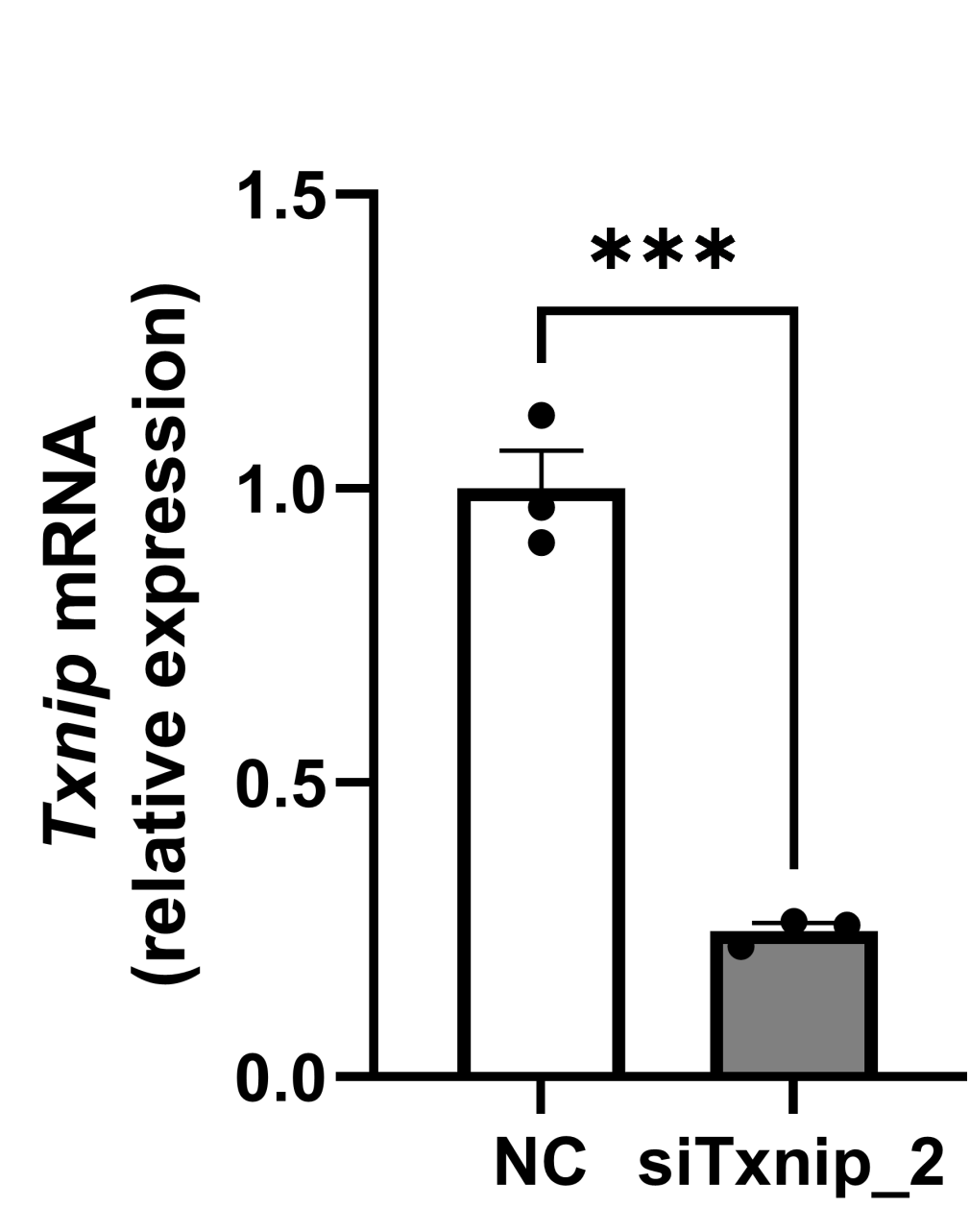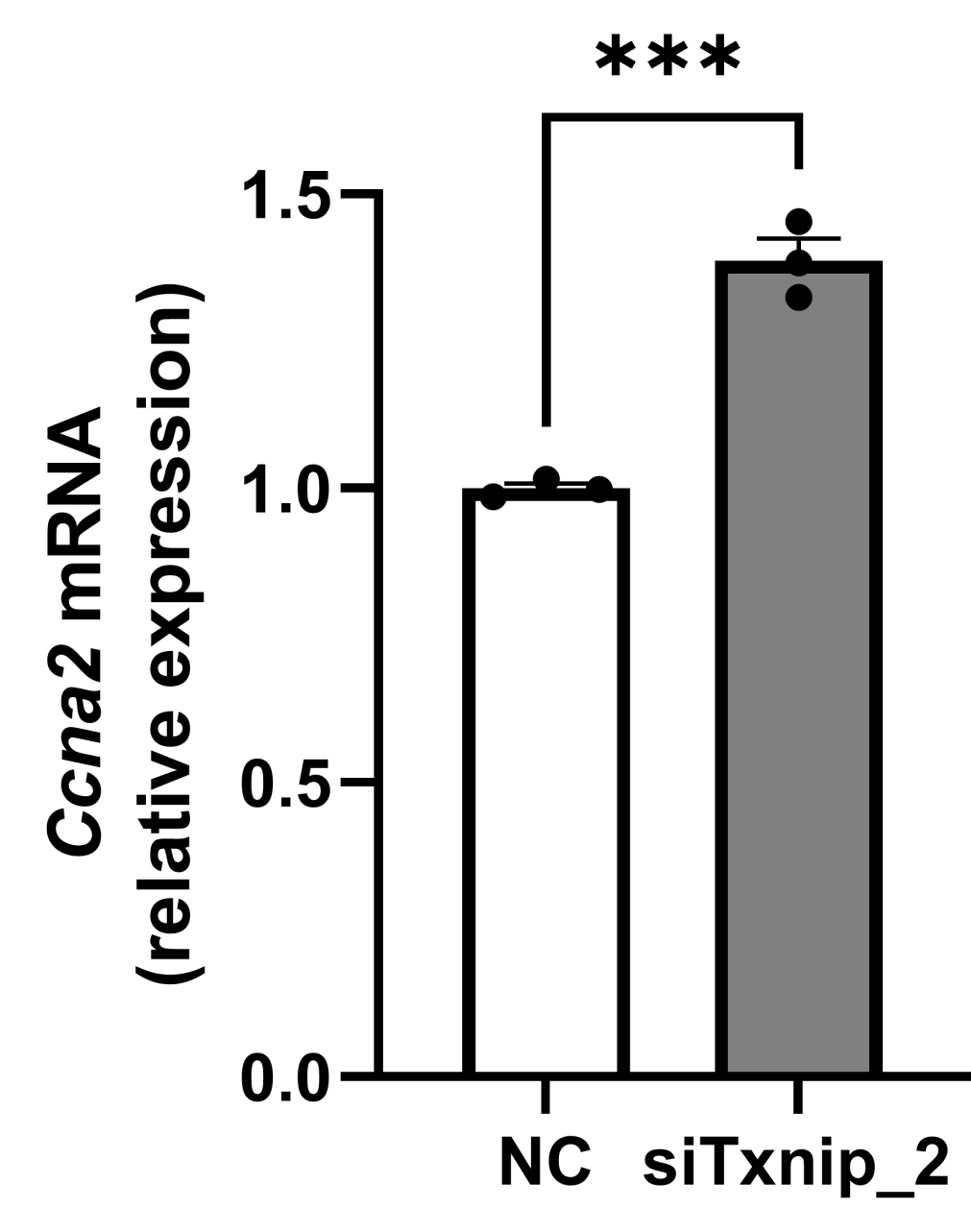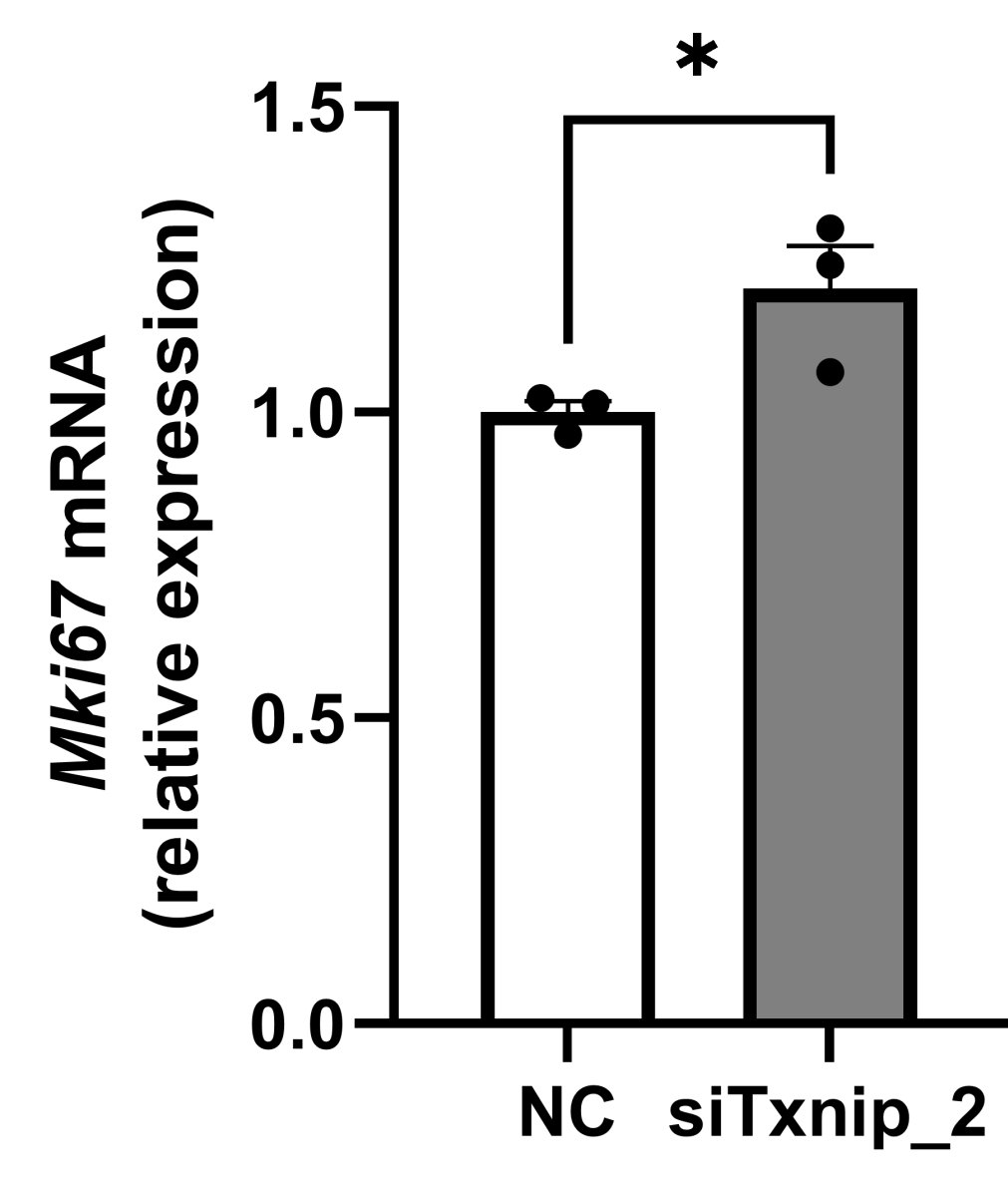

Supplement: S5 Fig — Txnip, Ccna2, and Mki67 mRNA levels of primary cultured hepatocytes transfected with 10 nM of scrambled or Txnip-targeting siRNA (siTxnip_2). *p < 0.05, ***p < 0.001. n = 3. (PDF) [file pone.0284989.s005.pdf]
