# Supplementary material for: Increased serum extracellular vesicle miR-144-3p and miR-486a-3p in a mouse model of adipose tissue regeneration promote hepatocyte proliferation by targeting Txnip
Source: PLoS One. 2023 May 4;18(5):e0284989. doi: 10.1371/journal.pone.0284989 (PMC10159167; doi:10.1371/journal.pone.0284989)
Supplement: S1 Table — (PDF) [file pone.0284989.s006.pdf]

**Gene**	**Primer sequence**
Adipoq	Fw : ATGGCAGAGATGGCACTCCT
	Rv : CCTTCAGCTCCTGTCATTCCA
Ccna2	Fw : GCCTTCACCATTCATGTGGAT
	Rv : TTGCTGCGGGTAAAGAGACAG
Ccnb1	Fw : AAGGTGCCTGTGTGTGAACC
	Rv : GTCAGCCCCATCATCTGCG
Cd34	Fw : AAGGCTGGGTGAAGACCCTTA
	Rv : TGAATGGCCGTTTCTGGAAGT
Cdk1	Fw : AGAAGGTACTTACGGTGTGGT
	Rv : GAGAGATTTCCCGAATTGCAGT
Cdk2	Fw : CCTGCTTATCAATGCAGAGGG
	Rv : TGCGGGTCACCATTTCAGC
Dpp4	Fw : ACCGTGGAAGGTTCTTCTGG
	Rv : CACAAAGAGTAGGACTTGACCC
Fabp4	Fw : AAGGTGAAGAGCATCATAACCCT
	Rv : TCACGCCTTTCATAACACATTCC
Insr	Fw : TCTGAGAAAGAGGCAGCCG
	Rv : GCACGTACACAGAAGATGGA
Lep	Fw : GAGACCCCTGTGTCGGTTC
	Rv : CTGCGTGTGTGAAATGTCATTG
Mki67	Fw : ATCATTGACCGCTCCTTTAGGT
	Rv : GCTCGCCTTGATGGTTCCT
Pi16	Fw : GGGGCCACAACAAAGAACG
	Rv : CACATCTGGTTCGGATCGCA
Txnip	Fw : TCTTTTGAGGTGGTCTTCAACG
	Rv : GCTTTGACTCGGGTAACTTCACA
