# Supplementary material for: Increased serum extracellular vesicle miR-144-3p and miR-486a-3p in a mouse model of adipose tissue regeneration promote hepatocyte proliferation by targeting Txnip
Source: PLoS One. 2023 May 4;18(5):e0284989. doi: 10.1371/journal.pone.0284989 (PMC10159167; doi:10.1371/journal.pone.0284989)
Supplement: S1 Raw images — (PDF) [file pone.0284989.s007.pdf]

Fig 2A

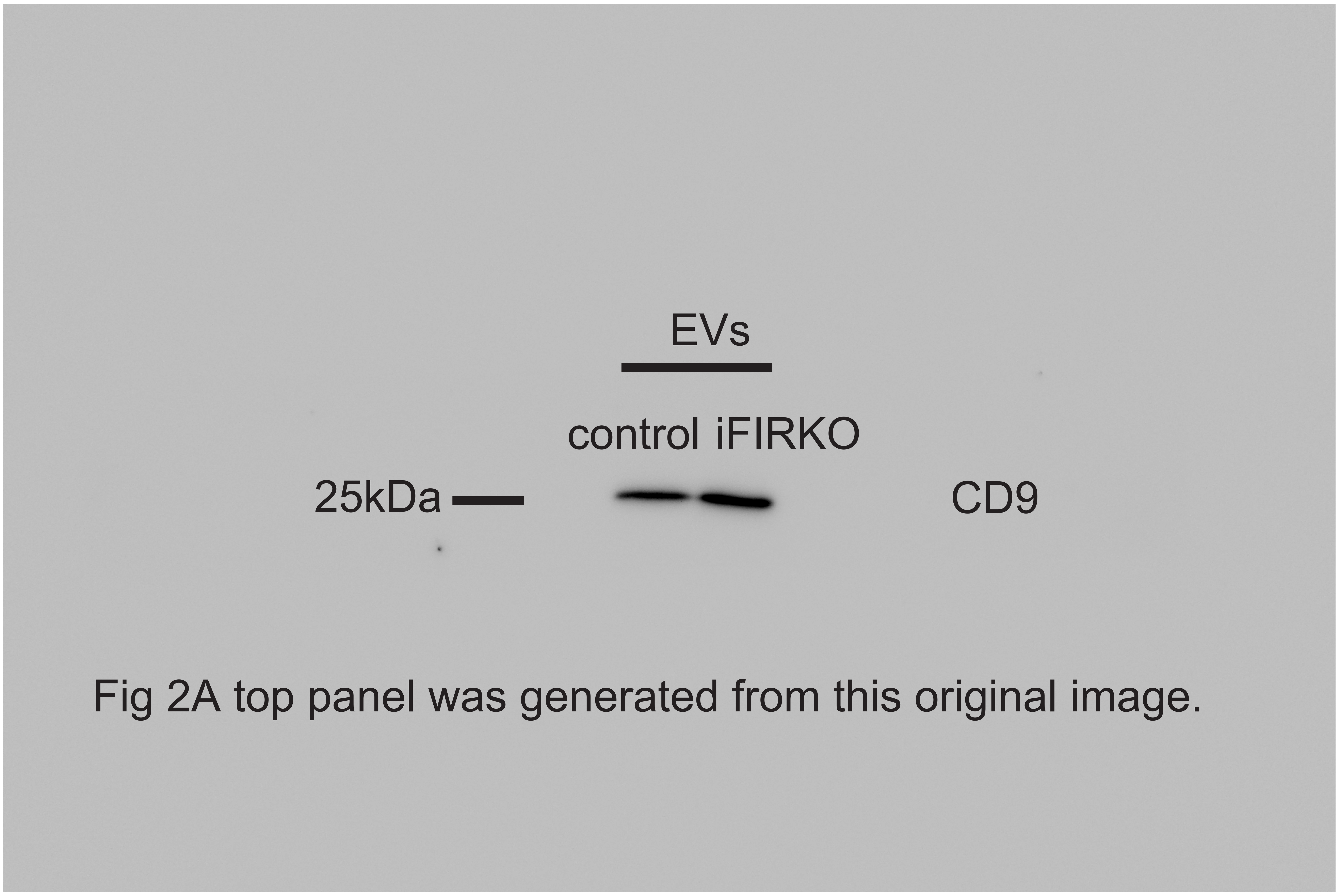

Fig 2A top panel was generated from this original image.

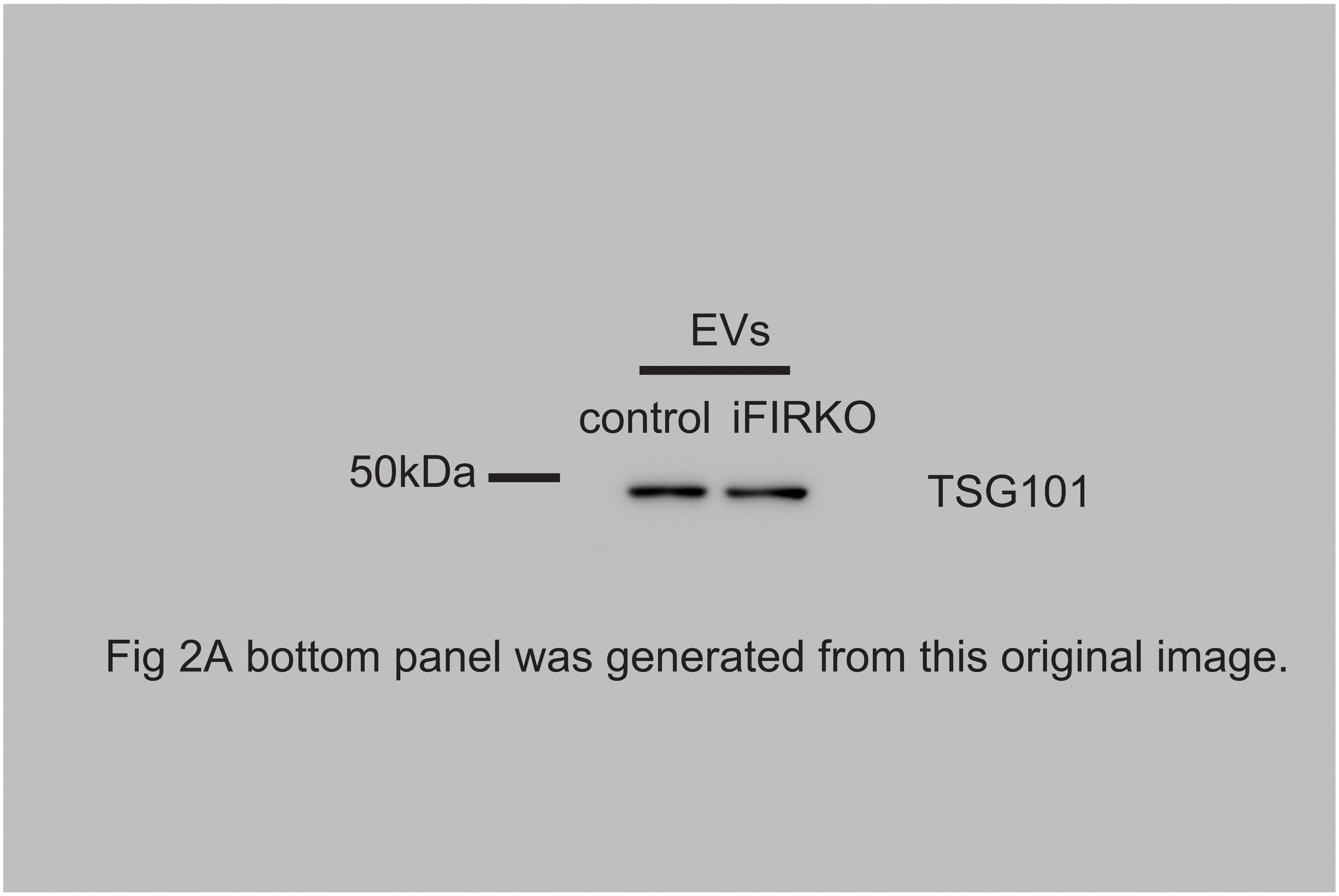

Fig 2A bottom panel was generated from this original image.

Fig 5B

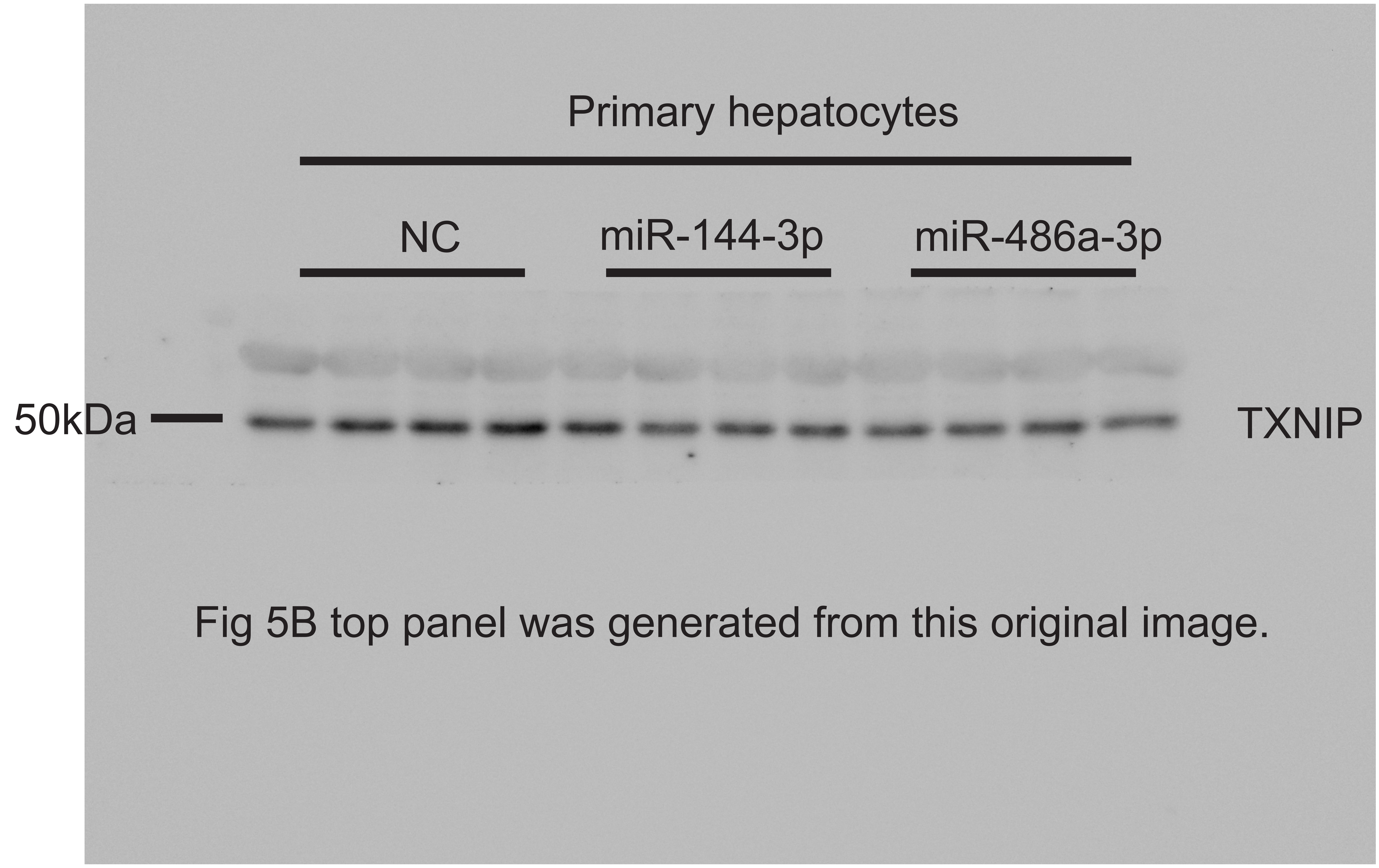

Fig 5B top panel was generated from this original image.

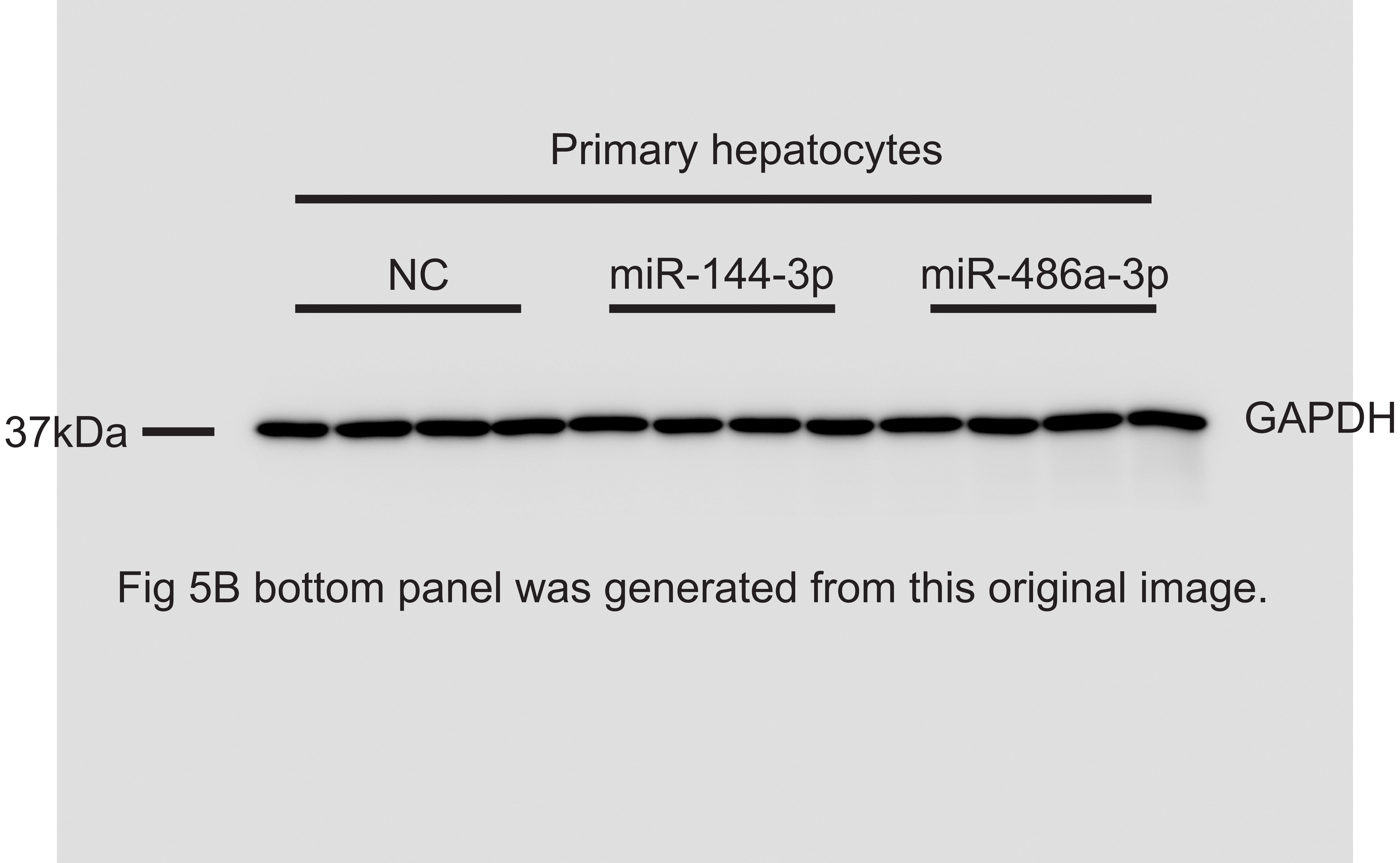

Fig 5B bottom panel was generated from this original image.

Fig 5C

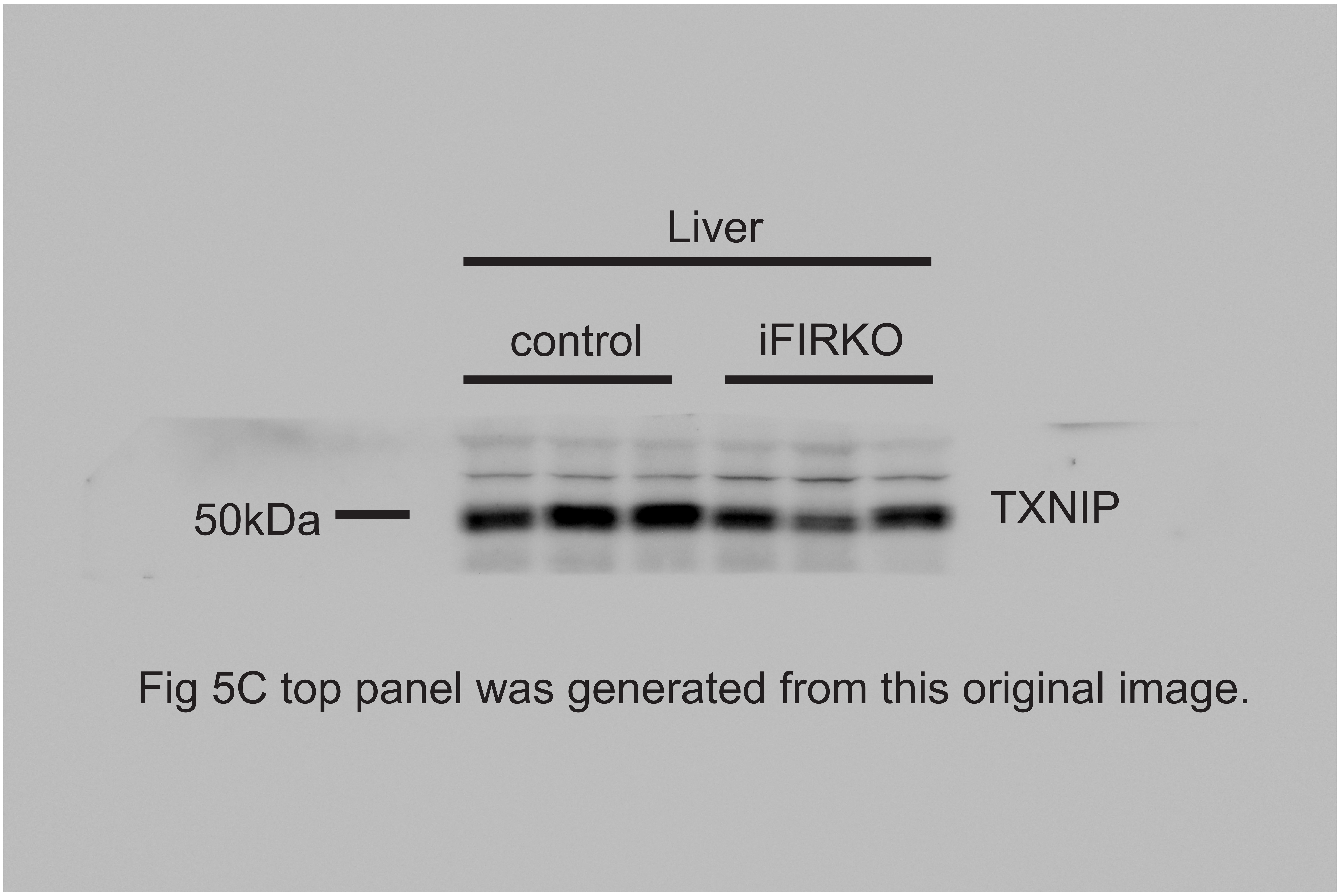

Fig 5C top panel was generated from this original image.

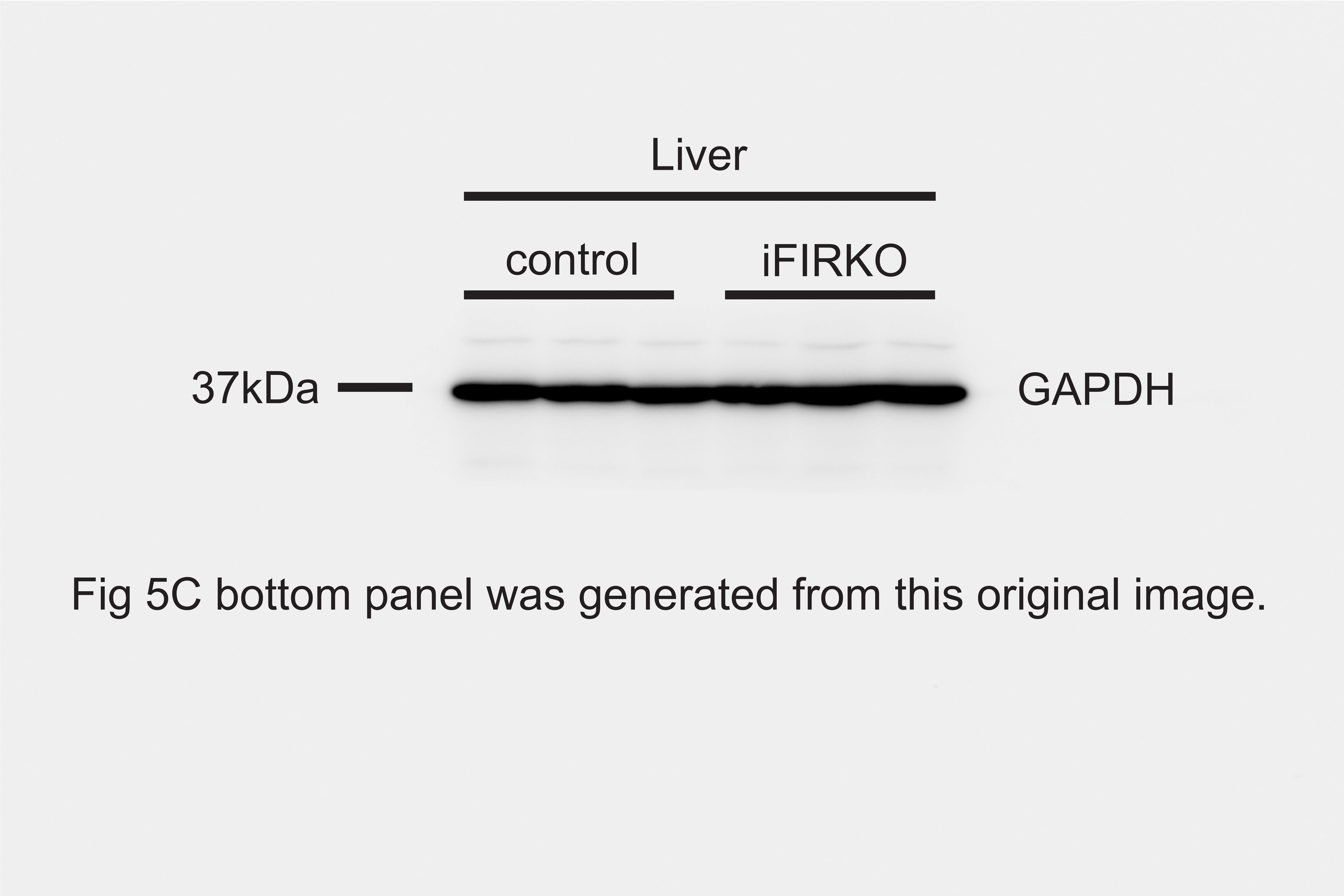

Fig 5C bottom panel was generated from this original image.
